# Heteroheptacene-based acceptors with thieno[3*,*2-*b*]pyrrole yield high-performance polymer solar cells

**DOI:** 10.1093/nsr/nwac076

**Published:** 2022-04-27

**Authors:** Zhenghui Luo, Ruijie Ma, Jianwei Yu, Heng Liu, Tao Liu, Fan Ni, Jiahao Hu, Yang Zou, Anping Zeng, Chun-Jen Su, U-Ser Jeng, Xinhui Lu, Feng Gao, Chuluo Yang, He Yan

**Affiliations:** Shenzhen Key Laboratory of Polymer Science and Technology, College of Materials Science and Engineering, Shenzhen University, Shenzhen 518060, China; Department of Chemistry and Hong Kong Branch of Chinese National Engineering Research Center for Tissue Restoration & Reconstruction, Hong Kong University of Science and Technology (HKUST), Hong Kong, China; Department of Chemistry and Hong Kong Branch of Chinese National Engineering Research Center for Tissue Restoration & Reconstruction, Hong Kong University of Science and Technology (HKUST), Hong Kong, China; Hong Kong University of Science and Technology-Shenzhen Research Institute, Shenzhen 518057, China; Department of Physics, Chemistry and Biology (IFM), Linköping University, Linköping SE-58183, Sweden; Department of Physics, Chinese University of Hong Kong, Hong Kong, China; Department of Chemistry and Hong Kong Branch of Chinese National Engineering Research Center for Tissue Restoration & Reconstruction, Hong Kong University of Science and Technology (HKUST), Hong Kong, China; Hong Kong University of Science and Technology-Shenzhen Research Institute, Shenzhen 518057, China; Shenzhen Key Laboratory of Polymer Science and Technology, College of Materials Science and Engineering, Shenzhen University, Shenzhen 518060, China; Shenzhen Key Laboratory of Polymer Science and Technology, College of Materials Science and Engineering, Shenzhen University, Shenzhen 518060, China; Shenzhen Key Laboratory of Polymer Science and Technology, College of Materials Science and Engineering, Shenzhen University, Shenzhen 518060, China; Department of Chemistry and Hong Kong Branch of Chinese National Engineering Research Center for Tissue Restoration & Reconstruction, Hong Kong University of Science and Technology (HKUST), Hong Kong, China; Hong Kong University of Science and Technology-Shenzhen Research Institute, Shenzhen 518057, China; Synchrotron Radiation Research Center, Hsinchu Science Park, Hsinchu 30076, China; Synchrotron Radiation Research Center, Hsinchu Science Park, Hsinchu 30076, China; Department of Chemical Engineering, Tsing Hua University, Hsinchu 30013, China; Department of Physics, Chinese University of Hong Kong, Hong Kong, China; Department of Physics, Chemistry and Biology (IFM), Linköping University, Linköping SE-58183, Sweden; Shenzhen Key Laboratory of Polymer Science and Technology, College of Materials Science and Engineering, Shenzhen University, Shenzhen 518060, China; Department of Chemistry and Hong Kong Branch of Chinese National Engineering Research Center for Tissue Restoration & Reconstruction, Hong Kong University of Science and Technology (HKUST), Hong Kong, China; Hong Kong University of Science and Technology-Shenzhen Research Institute, Shenzhen 518057, China; Hong Kong University of Science and Technology (HKUST) Light-Emitting Diode and Flat Panel Display Technology Research & Development Center, Foshan 526040, China; Hong Kong University of Science and Technology (HKUST) Foshan Research Institute for Smart Manufacturing, Hong Kong, China

**Keywords:** thieno[3,2-*b*]pyrrole, small-molecule acceptors, energy loss, intramolecular non-covalent interactions, polymer solar cells

## Abstract

Rationally utilizing and developing synthetic units is of particular significance for the design of high-performance non-fullerene small-molecule acceptors (SMAs). Here, a thieno[3*,*2-*b*]pyrrole synthetic unit was employed to develop a set of SMAs (ThPy1, ThPy2, ThPy3 and ThPy4) by changing the number or the position of the pyrrole ring in the central core based on a standard SMA of IT-4Cl, compared to which the four thieno[3*,*2-*b*]pyrrole-based acceptors exhibit bathochromic absorption and upshifted frontier orbital energy level due to the strong electron-donating ability of pyrrole. As a result, the polymer solar cells (PSCs) of the four thieno[3*,*2-*b*]pyrrole-based acceptors yield higher open-circuit voltage and lower energy loss relative to those of the IT-4Cl-based device. What is more, the ThPy3-based device achieves a power conversion efficiency (PCE) (15.3%) and an outstanding fill factor (FF) (0.771) that are superior to the IT-4Cl-based device (PCE = 12.6%, FF = 0.758). The ThPy4-based device realizes the lowest energy loss and the smallest optical band gap, and the ternary PSC device based on PM6:BTP-eC9:ThPy4 exhibits a PCE of 18.43% and a FF of 0.802. Overall, this work sheds light on the great potential of thieno[3,2-*b*]pyrrole-based SMAs in realizing low energy loss and high PCE.

## Teaser

Four heteroheptacene-based acceptors using thieno[3,2-*b*]pyrrole building block were developed for the first time, and all the four acceptors-based devices realized high performance and low energy loss.

## INTRODUCTION

Inclusion of non-fullerene small-molecule acceptors (SMAs) into polymer solar cells (PSCs) stimulates the power conversion efficiency (PCE) to new levels [1–3]. The remarkable characteristics of SMAs, such as easily modified molecular structure, strong light-absorption capacity and highly adjustable energy levels and optical band gaps, inspire novel molecular design strategies and synthetic methods to develop efficient non-fullerene SMAs, especially for acceptor–donor–acceptor (A–D–A)-type SMAs [4–6] to further boost the device performance of non-fullerene PSCs. The most commonly utilized means of tweaking the efficiency of SMAs include (i) enhancing the intramolecular charge-transfer (ICT) effect to broaden the absorption spectra of SMAs [7–9], (ii) increasing the energy level of the lowest unoccupied molecular orbital (LUMO) while maintaining a relatively red-absorbing spectra to reduce the energy loss (*E*_loss_) [10–12] and (iii) employing molecular asymmetric or isomerization synthetic strategies to tune the optoelectronic properties of SMAs [13–15].

In general, the A–D–A-type SMAs consist of an electron-donating central core, side chains and two electron-accepting terminal units [10–12,16], of which the fused-ring central cores are the most challenging and important tuning targets for boosting photovoltaic performance [17–19]. Although the PCEs of binary devices based on non-fullerene SMAs have exceeded 18%, we found that the central core of non-fullerene SMAs in these high-efficiency systems is relatively limited, especially for the outermost aromatic rings of the central core [20,21]. Choosing suitable outermost aromatic rings is particularly important for the design of high-efficiency SMAs because the direct electron delocalization between the outermost aromatic ring and the terminal unit has a great impact on the physicochemical characteristics of SMAs [22–26]. Commonly used outermost aromatic rings in A–D–A-type SMAs include thiophene, selenophene, thieno[3,2-*b*]thiophene, thieno[3,2-*b*]selenophene [27–30], dithieno[3*,*2-*b* : 2*^′^,*3*^′^*-*d*]thiophene [31–33] and dithieno[3*,*2-*b* : 2*^′^,*3*^′^*-d]pyrrole (DTP) [34–36]. In comparison with thiophene and selenophene, pyrrole is the most electron-rich five-membered single aromatic ring, which can enhance the ICT effect, reduce optical band gaps (*E*_g_^opt^) and increase the energy level of the LUMO of SMAs [37–39]. Additionally, fine-tuning the side chains on pyrrole can improve the molecular packing, the solubility and the mobility of SMAs. Similarly, the presence of tricyclic DTP with a pyrrole group endows desirable properties on SMAs, including a small *E*_g_^opt^ and a shallow LUMO value. To date, almost all nitrogen-containing outermost aromatic rings of SMAs are composed of monocyclic pyrrole or three-ring DTP. To our knowledge, there is no literature report on the use of bicyclic thieno[3,2-*b*]pyrrole (ThPy) as the synthetic unit to construct non-fullerene SMAs. The ThPy synthetic unit has received little attention in the entire field of organic electronics [40,41], which is probably due to its challenging synthesis.

**Figure 1.**
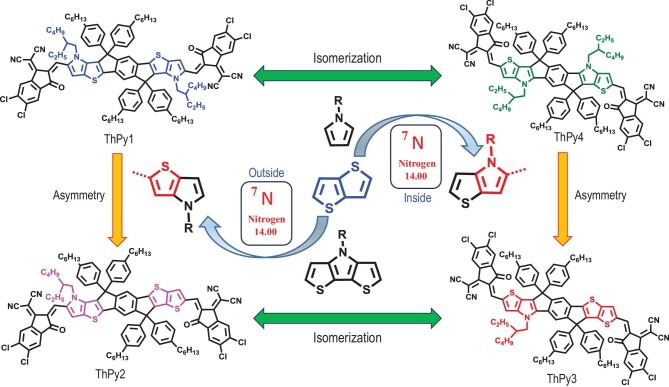
Molecular design strategy of ThPy1, ThPy2, ThPy3 and ThPy4.

In this study, we have systematically adjusted pyrrole substitution based on the famous SMA IT-4Cl [6] to develop the four SMAs, namely ThPy1, ThPy2, ThPy3 and ThPy4 (Fig. 1). Among them, ThPy1 and ThPy4 are a pair of isomers with centrosymmetric structures, and ThPy2 and ThPy3 are a second pair of isomers with an asymmetric central core. The difference between the two isomers in each pair is the position of the pyrrole ring in the central core: ThPy1 and ThPy2 have a pyrrole-exteriorized core, and ThPy3 and ThPy4 have a pyrrole-interiorized core. The number and positions of the pyrrole rings have a great impact on the band gap, energy levels, charge transport and molecular packing of the resulting SMAs. We found that the introduction of pyrrole rings in ThPy-based SMAs caused reduced band gaps and increased energy levels relative to those of IT-4Cl. When paired with the high-performance polymer donor PM6, the ThPy3-based device achieved a PCE of 15.3%, along with an open-circuit voltage (*V*_OC_) of 0.830 V, a short-circuit current density (*J*_SC_) of 23.82 mA cm^–2^, an outstanding fill factor (FF) (0.771) and a low energy loss (*E*_loss_) of 0.60 eV, which are superior to those of the IT-4Cl-based device (PCE = 12.6%, *V*_OC_ = 0.798 V, *J*_SC_ = 20.89 mA cm^–2^, FF = 0.758, *E*_loss_ = 0.74 eV). The improved FF in the ThPy3-based device is mainly attributed to its more balanced charge transport, weaker bimolecular recombination and better molecular packing. These results suggest that the thieno[3,2-*b*]pyrrole synthetic unit shows great prospects in the development of low-energy and high-performance non-fullerene polymer solar cells.

## RESULTS AND DISCUSSION

**Scheme 1.**
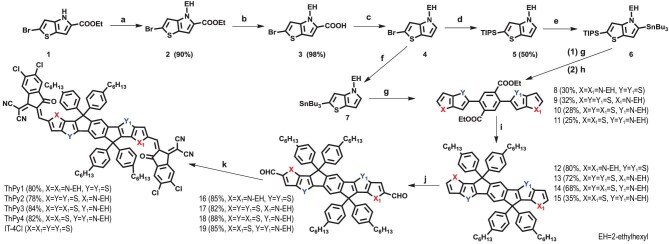
Synthetic route to ThPy1, ThPy2, ThPy3 and ThPy4. Reagents and conditions: (a) anhydrous K_2_CO_3_, 2-ethylhexyl bromide, DMF, 100°C, 12 h; (b) NaOH, EtOH/H_2_O [1 : 1 (v/v)]; (c) CH_3_COOAg, K_2_CO_3_, NMP, 150°C, 12 h; (d) (1) *n*-BuLi, THF, –78°C; (2) TIPSCl, –78°C to room temperature; (e) (1) *n*-BuLi, THF, 0°C; (2) Bu_3_SnCl, 0°C to room temperature; (f) (1) *n*-BuLi, THF, –78°C; (2) Bu_3_SnCl, –78°C to room temperature; (g) tributyl(thieno[3,2-*b*]thiophen-2-yl)stannane, 2,5-dibromo-terephthalic acid diethyl ester, PdCl_2_(PPh_3_)_2_, toluene, 110°C; (h) TBAF, THF, 0°C; (i) (1) 4-hexyl-1-bromobenzene, *n*-BuLi, THF, –78°C to room temperature; (2) HOAc, H_2_SO_4(conc)_, 100°C; (j) DMF, POCl_3_, 1,2-dichloroethane, 0°C to reflux; (k) 2-(5,6-dichloro-3-oxo-2,3-dihydro-1H-inden-1-ylidene)malononitrile, chloroform, pyridine, reflux 12 h.

The detailed synthetic route of ThPy1, ThPy2, ThPy3 and ThPy4 is presented in Scheme 1 and Scheme S1 in the Supporting Information (SI). The Starting material 1, obtained from commercially available 5-bromo-2-thiophenecarboxyaldehyde, ethyl azidoacetate and ethyl trifluoroacetate [40], underwent a nucleophilic substitution reaction with 2-ethylhexyl bromide to afford Compound 2. Subsequently, a simple hydrolysis reaction using sodium hydroxide was performed to generate Compound 3 quantitatively. Decarboxylation of Compound 3 was accomplished with the addition of silver acetate and potassium carbonate to yield the key intermediate 2-bromo-4-(2-ethylhexyl)-4*H*-thieno[3,2-*b*]pyrrole (4), which is purified by column chromatography. Because Compound 4 is extremely unstable in air, the hexane solution containing Compound 4 was utilized directly for the next reaction to gradually transform the compound into four ladder-type cores, including two symmetric isomer and two asymmetric isomer, which successively went through Vilsmeier–Haack reaction and Knoevenagel condensation reaction to produce the final products ThPy1, ThPy2, ThPy3 and ThPy4, respectively. Conventional characterizations, including ^1^H NMR, ^13^C NMR and mass spectrometry, were conducted to verify the molecular structures of the key intermediates and the four target products. ThPy1, ThPy2, ThPy3 and ThPy4 can be readily dissolved in commonly used organic solvents, including chloroform (CF), toluene (Tol) and chlorobenzene (CB).

**Figure 2.**
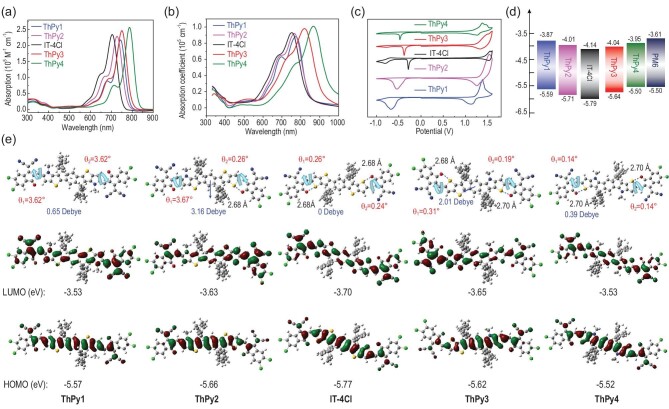
(a) Absorption spectra of ThPy1, ThPy2, ThPy3, ThPy4 and IT-4Cl in dilute CF solution; (b) film absorption spectra of ThPy1, ThPy2, ThPy3, ThPy4 and IT-4Cl; (c) CV curves of ThPy1, ThPy2, ThPy3, ThPy4 and IT-4Cl; (d) energy-level diagram of ThPy1, ThPy2, ThPy3, ThPy4, IT-4Cl and PM6; (e) optimal geometries, dipole moments and frontier molecular orbitals obtained using DFT for ThPy1, ThPy2, IT-4Cl, ThPy3 and ThPy4.

The optical properties of ThPy1, ThPy2, ThPy3, ThPy4 and IT-4Cl were surveyed, as shown in Fig. 2a and b, and the related data are outlined in Table S1 in the SI. In dilute CF solution, all acceptors exhibit a large maximum extinction coefficient (*ϵ*_max_) (for ThPy1, *ϵ*_max_ = 2.18 × 10^5^ M^−1^ cm^−1^ at 744 nm; for ThPy2, *ϵ*_max_ = 2.29 × 10^5^ M^−1^ cm^−1^ at 727 nm; for IT-4Cl, *ϵ*_max_ = 2.35 × 10^5^ M^−1^ cm^−1^ at 704 nm; for ThPy3, *ϵ*_max_ = 2.47 × 10^5^ M^−1^ cm^−1^ at 749 nm; for ThPy4, *ϵ*_max_ = 2.57 × 10^5^ M^−1^ cm^−1^ at 786 nm), indicating the strong light-absorbing capability of these acceptors. In comparison with IT-4Cl, the other four acceptors based on ThPy display red-shifted absorption spectra due to the strong electron-donating ability of the pyrrole ring. In addition, the pyrrole-interiorized isomers ThPy3 and ThPy4 possess a more strongly red-shifted absorption compared with the pyrrole-exteriorized isomers ThPy1 and ThPy2 due to the more favorable molecular characteristics. When prepared as films, bathochromic shifts of 28, 38, 48, 70 and 81 nm are observed for ThPy1, ThPy2, IT-4Cl, ThPy3 and ThPy4 pristine films, respectively. The larger red shifts of IT-4Cl, ThPy3 and ThPy4 may be related to better intermolecular π–π interactions (as discussed below) relative to those of ThPy1 and ThPy2. In the solid state, these five acceptors exhibit high and similar absorption coefficients of ∼0.95 × 10^5^ cm^−1^ (0.88 × 10^5^ cm^−1^ for ThPy1; 0.92 × 10^5^ cm^−1^ for ThPy2; 0.94 × 10^5^ cm^−1^ for IT-4Cl; 0.97 × 10^5^ cm^−1^ for ThPy3; and 1.01 × 10^5^ cm^−1^ for ThPy4), contributing to the large *J*_SC_ values. The *E*_g_^opt^s are estimated to be 1.46 eV for ThPy1, 1.47 eV for ThPy2, 1.49 eV for IT-4Cl, 1.35 eV for ThPy3 and 1.27 eV for ThPy4, respectively.

The electrochemical properties of these five acceptors were probed using cyclic voltammetry (CV) experiments (Fig. 2c and d). The onset oxidation and reduction potentials of ThPy1, ThPy2, IT-4Cl, ThPy3 and ThPy4 versus Ag/Ag^+^ were found to be 1.22/−0.50 eV, 1.34/−0.36 eV, 1.42/−0.23 eV, 1.27/−0.33 eV and 1.13/−0.42 eV, respectively. The measured relative energy level of Fc/Fc^+^ and the assumed vacuum energy level were 0.43 and –4.8 eV, respectively. Therefore, the highest occupied molecular orbital (HOMO) and LUMO energy levels are calculated to be –5.59/–3.87 eV for ThPy1, –5.71/–4.01 eV for ThPy2, –5.79/–4.14 eV for IT-4Cl, –5.64/–4.04 eV for ThPy3 and –5.50/–3.95 eV for ThPy4. The upshifted HOMO and LUMO values of ThPy-based acceptors, relative to those of IT-4Cl, should be attributed to the presence of the strongly electron-donating pyrrole. The upshifted LUMO energy levels are conducive to achieving high *V*_OC_. Compared with ThPy1 and ThPy2, the corresponding isomers ThPy4 and ThPy3 give a shallower HOMO value but a deeper LUMO value, which could be ascribed to the better communication and lower torsional angles between the central core and the indanone units, as the more intramolecular non-covalent interactions of S⋅⋅⋅O occur in ThPy4 and ThPy3.

Density functional theory (DFT) calculations were performed to investigate the energy levels, dipole moments and optimal geometries of the SMAs (Fig. 2e). The torsional angles are *θ*_1_ = 3.62^o^/*θ*_2_=3.62^o^ for ThPy1, *θ*_1_ = 3.67^o^/*θ*_2_ = 0.26^o^ for ThPy2, *θ*_1_ = 0.26^o^/*θ*_2_ = 0.24^o^ for IT-4Cl, *θ*_1_ = 0.31^o^/*θ*_2_ = 0.19^o^ for ThPy3 and *θ*_1_ = 0.14^o^/*θ*_2_ = 0.14^o^ for ThPy4, suggesting that the intramolecular non-covalent interactions of S⋅⋅⋅O can improve molecular planarity, which is beneficial to enhancing molecular packing and promoting charge mobility. The distance between the O in the indanone unit and the S in the outermost thiophene of the central core is ∼2.70 Å for ThPy2, IT-4Cl, ThPy3 and ThPy4, confirming the existence of S···O intramolecular non-covalent interactions. The asymmetric isomers ThPy2 and ThPy3 yield significantly larger dipole moments relative to those of the symmetric molecules ThPy1, IT-4Cl and ThPy4, consistently with our previous results. The calculated energy levels of these five acceptors exhibit the same trend as observed in the data from the CV method, which once again indicates that the electron richness of the pyrrole ring raises the energy levels of the frontier orbitals.

**Figure 3.**
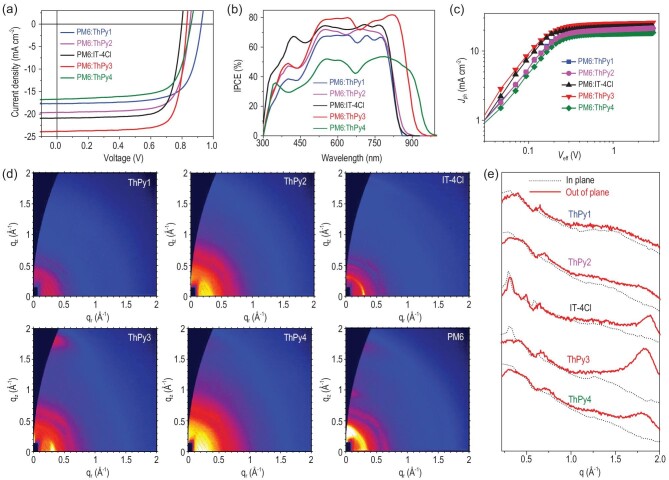
(a) *J*–*V* characteristics of the best PSCs under the illumination of AM 1.5G, 100 mW cm^−2^; (b) EQE spectra; (c) *J*_ph_ versus *V*_eff_ of the optimal PSC devices; (d) GIWAXS patterns of ThPy1, ThPy2, IT-4Cl, ThPy3, ThPy4 and PM6 neat films; (e) corresponding intensity profiles of ThPy1, ThPy2, IT-4Cl, ThPy3 and ThPy4 neat films along the in-plane (in black) and out-of-plane (in red) direction.

The impact of pyrrole substitution on the device efficiency was probed by matching these five acceptors with the high-performance polymer donor PM6, which exhibits an absorption spectrum that is highly complementary with those of the SMAs. These devices were prepared using a conventional configuration of indium tin oxide (ITO)/PEDOT : PSS/PM6 : acceptors/PNDIT-F3N/Ag. Herein, PEDOT : PSS (poly(3,4-ethylenedioxy-thiophene) : poly(styrene-sulfonate)) and PNDIT-F3N ((9,9-bis(3*^′^*-(*N*,*N*-dimethylamino)propyl)-2,7-fluorene)-alt-5,5*^′^*-bis(2,2*^′^*-thiophene)-2,6-naph-thalene-1,4,5,8- tetracaboxylic-*N*,*N^′^*-di(2-ethylhexyl)imide)) were utilized as the hole-transporting layer and the electron-transporting layer, respectively. The fabrication process of the best-performing device was provided in the SI. The optimal current density–voltage (*J*–*V*) curves and the corresponding external quantum efficiency (EQE) spectra are illustrated in Fig. 3a and b, and the related key photovoltaic data are outlined in Table 1.

**Table 1.** Photovoltaic data of the PM6:acceptor-based PSCs, and in parentheses are average values based on 10 devices.

Devices	*V*_OC_ (V)	*J*_SC_ (mA cm^–2^)	FF	PCE_max_ (PCE_avg_) %
				
ThPy1	0.924	17.68	0.711	11.6 (11.27 ± 0.22)
ThPy2	0.850	19.63	0.738	12.3 (11.92 ± 0.24)
IT-4Cl	0.798	20.89	0.758	12.6 (12.45 ± 0.16)
ThPy3	0.830	23.82	0.771	15.3 (14.90 ± 0.24)
ThPy4	0.857	16.70	0.701	10.0 (9.78 ± 0.12)
BTP-eC9	0.847	26.41	0.783	17.51 (17.16 ± 0.17)
BTP-eC9^a^	0.849	27.08	0.802	18.43 (18.18 ± 0.19)
BTP-eC9^b^	0.849	26.36	0.791	17.70 (17.35 ± 0.23)

^a^PM6 : BTP-eC9 : ThPy4 (1 : 1 : 0.1) device; ^b^PM6 : BTP-eC9 : ThPy4 (1 : 1 : 0.2) device.

Basically, the PM6 : IT-4Cl device delivers a PCE of 12.6%, with a *V*_OC_ of 0.798 V, a *J*_SC_ of 20.89 mA cm^–2^ and an FF of 0.758, which is comparable to our previously reported results. When the thiophene was replaced by 1-(2-ethylhexyl)-1H-pyrrole, the devices constructed from ThPy-based SMAs realize significantly enhanced *V*_OC_s (0.924 V for ThPy1, 0.850 V for ThPy2, 0.830 V for ThPy3 and 0.857 V for ThPy4) because of the upshifted LUMO energy levels. In comparison with symmetric ThPy-based SMAs, the asymmetric counterparts give significantly increased values of *J*_SC_ and FF, and thus better PCE. As a result, the ThPy3-based device achieves the best PCE of 15.3% among these five devices, with the highest *J*_SC_ of 23.82 mA cm^–2^ and the largest FF of 0.771. The PM6 : ThPy4 device yields the worst PCE of 10.0% because it has the lowest *J*_SC_ of 16.70 mA cm^–2^ and FF of 0.701, which could originate from the mismatched energy levels between PM6 [17] and ThPy4. As shown in Fig. 3b, the ThPy3-based device exhibits stronger EQE responses in the wavelength range of 500–870 nm than the other devices containing ThPy-based SMAs. The remarkably broader EQE response in the ThPy3-based device explains its larger *J*_SC_ value relative to that of the IT-4Cl-based device. The integrated *J*_SC_ values of ThPy1-, ThPy2-, IT-4Cl-, ThPy3- and ThPy4-based cells from their EQE spectra in the same range are 17.31, 18.95, 20.35, 23.58 and 16.20 mA cm^−2^, respectively, matching well with the measured *J*_SC_ values.

To gain insight into the properties of charge extraction and exciton dissociation in the five best-performing PSCs, the curves of photocurrent density (*J*_ph_) versus effective voltage (*V*_eff_) are drawn in Fig. 3c and the corresponding parameters are listed in Table S2. The *J*_ph_ approaches saturation (*J*_sat_) when the *V*_eff_ exceeds 2V. The ratios of *J*_ph_/*J*_sat_ represent charge collection efficiency (*P*_coll_) and exciton dissociation efficiency (*P*_diss_) under the maximum power output and short-circuit conditions, respectively. The *P*_diss_/*P*_coll_ values are 91.6%/77.0% for the ThPy1-based device, 92.1%/79.1% for the ThPy2-based device, 93.1%/82.6% for the IT-4Cl-based device, 93.2%/85.0% for the ThPy3-based device and 90.8%/73.9% for the ThPy4-based device, implying the best charge collection and exciton dissociation in the PM6 : ThPy3 device, which coincides with its higher EQE and *J*_SC_ values.

To clarify the effect of the number and the position of pyrrole rings on charge-transporting behaviors, the charge mobilities in PM6 : SMA blends were investigated by performing space-charge-limited current experiments (Fig. S1 in the SI). As listed in Table S3, compared with the blend film of symmetric ThPy-based SMAs and PM6, the asymmetric counterpart-based blends exhibit larger hole (*μ*_h_) and electron (*μ_e_*) mobilities and more balanced *μ*_h_/*μ_e_*, which is consistent with the enhanced FF values of the symmetric structures compared to those of the asymmetric ones. The PM6 : ThPy3 device shows the largest hole mobility of 8.82 × 10^–4^ cm^2^ V^–1^ s^–1^ and electron mobility of 5.51 × 10^–4^ cm^2^ V^–1^ s^–1^ and a ratio of *μ*_h_/*μ_e_* that is closest to 1, suggesting the best charge-transport properties in the PM6 : ThPy3 device, which is beneficial for producing high *J*_SC_ and FF values.

**Figure 4.**
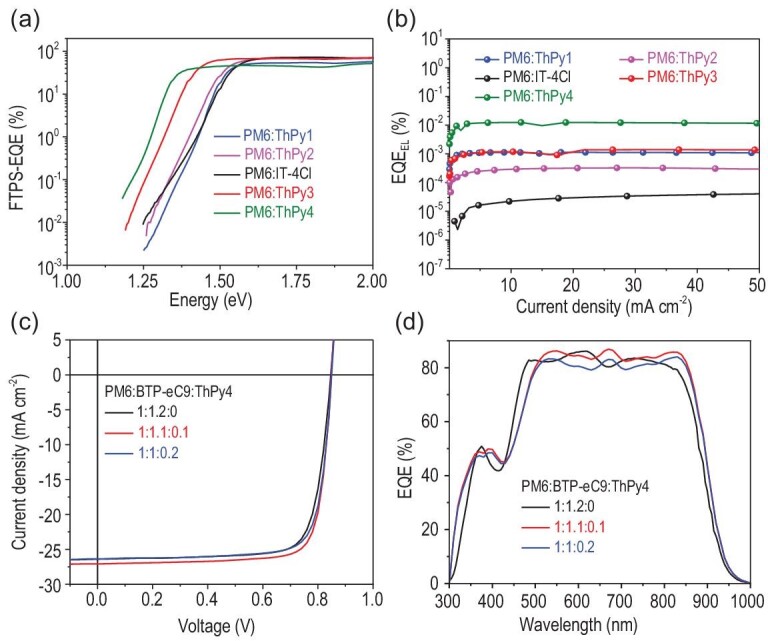
(a) FTPS–EQE spectra; (b) EL quantum efficiencies of the cells at various injection current densities; (c) *J–V* curves of the PM6 : BTP-eC9-based binary device and the PM6 : BTP-eC9 : ThPy4-based ternary device; (d) EQE spectra of the corresponding ternary device.

In addition to charge extraction and transport behaviors, the morphology of the active layer is of particular importance for the device performance. Grazing incident wide-angle X-ray scattering experiments were conducted to study the solid-state molecular orientation and crystallinity of neat acceptor and blend films [42–44]. As displayed in Fig. 4, ThPy1 and ThPy2 do not show obvious diffraction signals in the in-plane (IP) direction or in the out-of-plane (OOP) direction, implying their very weak crystalline properties. On the contrary, the interior N-substituted ThPy3 and ThPy4 SMAs yield significant diffraction signals, with the (100) diffraction peak at ∼0.31 Å^–1^ in the IP direction and the (010) π–π stacking peak at ∼1.83 Å^–1^ in the OOP direction, because of the more non-covalent interaction of O···S. IT-4Cl, ThPy3 and ThPy4 neat films present a predominantly face-on orientation, especially for ThPy3, which contributes to the vertical charge transport. In addition, ThPy3 shows the highest scattering intensity and the largest peak area in the OOP direction relative to those of IT-4Cl and ThPy4, indicating the better crystallinity of ThPy3. In blends of each of the five acceptors with PM6, all five exhibit similar crystal coherence lengths (CCLs) (∼24 Å^–1^) in the OOP direction. The intensities of the characteristic diffraction peaks of these blends were significantly changed from those of the SMAs; these intensities could be influenced by the presence of the polymer donor PM6. Similar strong (100) peaks at ∼0.31 Å^–1^ in the IP direction were observed (Fig. S2), along with the resulting CCL of PM6 : ThPy1, PM6 : ThPy2, PM6 : IT-4Cl, PM6 : ThPy3 and PM6 : ThPy4 films of 62.8, 56.2, 51.3, 96.8 and 72.4 Å, respectively. The larger CCL of PM6 : ThPy3 in the IP direction resulted in a better crystalline quality and enhanced molecular packing, coinciding with the occurrence of the highest FF value in the ThPy3-based device. Atomic force microscopy (AFM) measurements were carried out to study the surface morphology of the five active layers. As illustrated in Fig. S3, all blend films exhibit smooth surfaces and the root mean square roughness values of the PM6 : ThPy1, PM6 : ThPy2, PM6 : IT-4Cl, PM6 : ThPy3 and PM6 : ThPy4 blend films are 1.28, 1.57, 2.98, 3.66 and 2.66 nm, respectively; these different values resulted from the different crystal morphologies of the five SMAs. In the AFM phase images, all five blends possess fibril-like bi-continuous networks, which is advantageous to charge transport.

To gain insight into the reasons for the higher *V*_OC_s in the devices of ThPy-based acceptors relative to that of the IT-4Cl-based device, Fourier transform photocurrent spectroscopy-EQE and electroluminescence (EL) experiments were performed to investigate the detailed *E*_loss_ components (*E*_loss_ = Δ*E*_1_ + Δ*E*_2_ + Δ*E*_3_) [45,46]. The first component (Δ*E*_1_ = *E*_gap_ – *q*Δ*V*_OC_^SQ^) measures the difference between the bandgap and *qV*_OC_ in the Shockley–Queisser limit. As shown in Table S4 and Fig. 4, these five devices gave a similar Δ*E*_1_ (0.26 ∼ 0.27 eV). The second component, radiative recombination loss (Δ*E*_2_ = *q*Δ*V*_OC_^rad; below gap^), comes from radiative recombination loss below the bandgap. In this work, Δ*E*_2_ was calculated to be 0.04, 0.06, 0.08, 0.05 and 0.02 eV for the devices based on ThPy1, ThPy2, IT-4Cl, ThPy3 and ThPy4, respectively, according to the different values of Δ*V*_OC_^rad;below gap^. The third component (Δ*E*_3_ = *q*Δ*V*_OC_^non-rad^* = *–*k*Tln(EQE_EL_)) stems from non-radiative recombination loss, which can be obtained from the EQE_EL_ values; the Δ*E*_3_ is a main factor in determining the energy loss. To calculate the *E*_loss_ more accurately, the band gaps (*E*_g_^PV^) of these devices are extracted to be 1.52, 1.51, 1.54, 1.42 and 1.34 eV for ThPy1, ThPy2, IT-4Cl, ThPy3 and ThPy4, respectively. The reduced HOMO offsets between PM6 and the four ThPy-based acceptors, compared with the offsets in IT-4Cl, could effectively decrease the energetic offsets between the local excited (LE) state and the charge-transfer (CT) state in the blends, due to the strong electron-donating ability of the pyrrole ring. The reduced offsets are beneficial for the formation of the LE–CT hybridization and the thermal population. Therefore, the presence of the pyrrole ring can suppress the non-radiative recombination loss from the CT state, thereby leading to a higher EQE_EL_ and a smaller non-radiative recombination loss in the blends [47]. The *E*_loss_ of 0.51 eV obtained for the ThPy4-based device is one of the smallest values observed in PSCs in the literature [2,4], implying the great potential of thieno[3,2-*b*]pyrrole-based SMAs in realizing ultra-low *E*_loss_.

Furthermore, we evaluated the potential of the four ThPy-based SMAs in ternary PSCs. Related data are summarized in Table 1 and Table S5. As expected, the addition of ThPy4 into PM6 : BTP-eC9 [48] can not only broaden the absorption spectrum to improve *J*_SC_, but also ensure that the open-circuit voltage does not decrease; these effects are helpful for enhancing PCE. The device based on PM6 : BTP-eC9 : ThPy4 (1 : 1.1 : 0.1) yields a PCE as high as 18.43%, with a *V*_OC_ of 0.849, a *J*_SC_ of 27.08 mA/cm^2^ and an impressive FF of 0.802, higher than that of the binary device based on PM6 : BTP-eC9 (PCE = 17.51%) (Fig. 4c). At an increased ratio of ThPy4 in BTP-eC9 : ThPy4, the FF and *J*_SC_ decrease, thereby reducing the PCE. However, employing the other three ThPy-based molecules as guest acceptors into the PM6 : BTP-eC9 host system reduced the device efficiency even when the mass ratio of the ThPy-based molecules was 0.1, which mainly results from the decreased *J*_SC_. From the view of material selection of a ternary system, ThPy3 shows deeper LUMO energy levels and blue-shifted absorption relative to those of BTP-eC9; therefore, the lower PCE of 17.31% in the PM6 : BTP-eC9 : ThPy3 ternary device is reasonable and predictable. The *J*_SC_ integrated from the EQE spectrum of 26.6 mA cm^−2^ for the optimal PM6 : BTP-eC9 : ThPy4 ternary device matches well with the result from the *J*–*V* curves (Fig. 4d). These results indicate the great potential of ThPy4 in achieving a high-performance ternary system.

## CONCLUSION

In summary, we developed four new SMAs (ThPy1, ThPy2, ThPy3 and ThPy4) using a thieno[3*,*2-*b*]pyrrole synthetic unit for the first time, by changing the number or the position of the pyrrole ring in the central cores based on IT-4Cl. Comparison of IT-4Cl and the four thieno[3*,*2-*b*]pyrrole-based acceptors reveals the importance of rational modification of the molecular structure to fine-tune the photoelectric properties and the photovoltaic performance of films containing SMAs. Replacing thiophene of IT-4Cl with pyrrole can significantly increase the HOMO/LUMO energy levels and reduce the optical band gaps. The effects on intermolecular interactions from varying the positions of the nitrogen atoms in the ThPy1/ThPy4 and the ThPy2/ThPy3 isomer pairs indeed influence the molecular planarity, energy levels, π–π stacking and charge transport. The ThPy3-based device yields a high PCE of 15.3% as compared with the IT-4Cl-based device, which mainly results from the increased energy level of the LUMO and the significantly red-shifted absorption spectra of ThPy3. Compared with the other three ThPy-based devices, the more favorable characteristics of the ThPy3-based device are mainly attributed to the suitable energy levels, significantly enhanced crystallinity, improved molecular packing, the most balanced *μ*_h_/*μ*_e_ and highest charge collection efficiency. The asymmetric ThPy-based SMAs show bigger dipole moments, higher charge transport properties and better molecular packing, and thus a larger FF, as compared with their symmetric counterparts. Importantly, the ternary PSC device based on PM6 : BTP-eC9 : ThPy4 (1.0 : 1.1 : 0.1) exhibits a PCE of 18.43% and a FF of 0.802, higher than those of the PM6 : BTP-eC9 binary device. Our work demonstrates an effective strategy to develop efficient SMAs by using a nonconventional thieno[3*,*2-*b*]pyrrole building block. We believe that our findings will encourage the search for more unconventional building blocks that can be used toward the realization of high-performing, low-energy-loss non-fullerene PSCs.

## METHODS

### Materials

PM6 and PNDIT-F3N were bought from Solarmer Materials Inc. and the detailed synthesis of the four ThPy-based acceptors is described in the Supplementary Data online.

### Device fabrication

A conventional device structure of ITO/PEDOT:PSS/active layer/PNDIT-F3N/Ag was employed to prepare organic solar cells. The ITO substrates were cleaned according to standard procedures and then treated using an ultraviolet ozone generator for 30 min. A thin layer of PEDOT : PSS was spin-coated onto the top of a clean substrate at 4000 r/min for 30 s and annealed subsequently at 150°C for 15 min. All the chloroform solutions (the ratio of D/A is 1 : 1; the donor concentration was 7 mg mL^–1^) with 0.25% vol 1,8-diiodooctane (DIO) as additive were stirred in a glove box for 12 h and then spin-coated on the PEDOT : PSS layer at 3000 r/min for 30 s after being kept on a 65°C hotplate. Then, the active layers were annealed at 90°C for 5 min. A thin PNDIT-F3N layer was spin-coated on the top of the active layers, followed by 100 nm Ag via thermally deposition. The Bruker Dektak XT stylus profilometer was utilized to probe the optimal thickness of active layers; the optimal thickness was ∼105 nm.

### *J–V* measurements

Keithley 2400 under AM 1.5 G illumination at 100 mW cm^−2^ a with Newport solar simulator was used to probe the current *J*–*V* curves of all the encapsulated solar cells. A standard Si solar cell with a KG5filter was applied to calibrate the light intensity and the device area of 5.9 mm² was measured using an Olympus BX51 optical microscope.

### EQE measurements

An Enlitech QE-S Solar Cell Spectral Response (Newport 300W lamp) system was utilized to measured EQEs.

## Supplementary Material

nwac076_Supplemental_File
