# Supplementary material for: Heteroheptacene-based acceptors with thieno[3,2-b]pyrrole yield high-performance polymer solar cells
Source: Natl Sci Rev. 2022 Apr 27;9(7):nwac076. doi: 10.1093/nsr/nwac076 (PMC9273303; doi:10.1093/nsr/nwac076)
Supplement: nwac076_Supplemental_File [file nwac076_supplemental_file.doc]

**Supporting Information**

**Heteroheptacene-Based Acceptors with Thieno[3*,*2-*b*]pyrrole Yield High-Performance Polymer Solar Cells**

Zhenghui Luo,^1,2^**^+^*** Ruijie Ma,^2,3^**^+^** Jianwei Yu,^4^**^+^**Heng Liu,^5^ Tao Liu,^2,3^* Fan Ni,^1^ Jiahao Hu,^1^ Yang Zou,^1^ Anping Zeng,^2,3^ Chun-Jen Su,^6^ U-Ser Jeng,^6,7^ Xinhui Lu,^5^ Feng Gao,^3^ Chuluo Yang,^1^* He Yan^2,3,8,9^*

^1^Shenzhen Key Laboratory of Polymer Science and Technology, College of Materials Science and Engineering, Shenzhen University, Shenzhen 518060, China. E-mail: [zhhuiluo@163.com](mailto:zhhuiluo@163.com), clyang@szu.edu.cn.

^2^Department of Chemistry and Hong Kong Branch of Chinese National Engineering Research Center for Tissue Restoration & Reconstruction, Hong Kong University of Science and Technology (HKUST), Hong Kong 999077, China. E-mail: liutao851505@126.com, [hyan@ust.hk](mailto:hyan@ust.hk).

^3^Hong Kong University of Science and Technology-Shenzhen Research Institute, Shenzhen 518057, China.

^4^Department of Physics, Chemistry and Biology (IFM), Linköping University, Linköping SE-58183, Sweden

^5^Department of Physics, Chinese University of Hong Kong, Hong Kong 999077, China.

^6^Synchrotron Radiation Research Center, Hsinchu Science Park, Hsinchu 30076, China.

^7^Department of Chemical Engineering, Tsing Hua University, Hsinchu 30013, China.

^8^Hong Kong University of Science and Technology (HKUST) Light-Emitting Diode and Flat Panel Display Technology Research & Development Center, Foshan 526040, China.

^9^Hong Kong University of Science and Technology (HKUST) Foshan Research Institute for Smart Manufacturing, Hong Kong 999077, China.

[**^+^**] These authors contributed equally to this work.

**Materials and Measurements**

All solvents and reagents were used as received from commercial sources and used without further purification unless otherwise specified. ^1^H NMR (400 MHz) and ^13^C NMR (100 MHz) spectra were measured on a MERCURYVX400 spectrometers. Mass spectra were recorded on a Shimadzu spectrometer. UV-vis-NIR absorption spectra were recorded on a Shimadzu UV-2700 recording spectrophotometer. Cyclic voltammetry (CV) measurements were carried out on a CHI voltammetric analyzer at room temperature. Tetrabutylammonium hexafluorophosphate (*n*-Bu_4_NPF_6_, 0.1 M) was used as the supporting electrolyte. The conventional three-electrode configuration consists of a platinum working electrode with a 2 mm diameter, a platinum wire counter electrode, and an Ag/AgCl wire reference electrode. Cyclic voltammograms were obtained at a scan rate of 100 mV/s. The film morphology was measured using an atomic force microscope (AFM, Bruker Dimension ICON) using the tapping mode. The RMS values of the surface AFM images are averaged based on five times testing on different areas for each sample. DFT calculations were performed by using Gaussian at the B3LYP-D3(BJ)/def2-SVP level, and the long alkyl chain was simplified as methyl. Compound 1 were synthesized according to the reported literature.^[1]^

**Mobility Measurements**

Hole and electron mobilities were measured using the space charge limited current (SCLC) method. Device structures are ITO/PEDOT:PSS/active layer/MoOx/Ag for hole-only devices and ITO/ZnO/active layer/PNDIT-F3N/Ag for electron-only devices. The SCLC mobilities were calculated by MOTT-Gurney equation: *J* = 9*ε*_0_*ε*_r_*μV^2^*/8*L^3^*. Where *J* is the current density, ε_r_ is the relative dieletiric constant of active layer material usually 2-4 for organic semiconductor, herein we use a relative dielectric constant of 3, *ε*_0_ is the permittivity of empty space, *µ* is the mobility of hole or electron and L is the thickness of the active layer, V is the internal voltage in the device, and *V* = *V*_Applied_ – *V*_Built-in_ (in the hole-only and the electron-only devices, the *V*_bi_ values are 0.2 V and 0 V respectively), where *V*_Applied_ is the voltage applied to the device, and *V*_Built-in_ is the built-in voltage resulting from the relative work function difference between the two electrodes.

**GIWAXS measurement**

GIWAXS measurement were carried out at the 23A SWAXS beamline at NSRRC, Hsinchu, with 10 keV beam and a Pilatus 1MF detector. The incidence angle is 0.2°. The samples for GIWAXS measurements were fabricated on silicon substrates using the same recipe for the device.

**FTPS-EQE, EQE_EL_ and EL Characterizations**

FTPS-EQE was measured using Vertex 70 from Bruker Optics, equipped with a quartz tungsten halogen lamp, quartz beam splitter and external detector option. A low-noise current amplifier (SR570) was used to amplify the photocurrent produced on illumination of the photovoltaic devices with light modulated by the Fourier transform infrared spectroscope (FTIR). The output voltage of the current amplifier was fed back into the external detector port of the FTIR, to be able to use the FTIR’s software to collect the photocurrent spectrum.

EQE_EL_ values were obtained from an in-house-built system including a Hamamatsu silicon photodiode 1010B, a Keithley 2400 SourceMeter to provide voltage and record injected current, and a Keithley 485 Picoammeter to measure the emitted light intensity.

EL spectra were measured using a light guide positioned close to the sample. The bias of EL measurement was applied on the devices using a Keithley 2400 Source Meter. The detector was a Newton EM-CCD Si array detector at -60 ºC with a Shamrock SR-303i spectrograph from Andor Tech.

**Scheme S1.** Synthesis of ThPy1, ThPy2, ThPy3 and ThPy4.

**Synthesis of Compound 2:**

To a solution of compound **1** (2.74 g, 10 mmol) and potassium carbonate (4.15 g, 30 mmol) in 20 mL N, N-dimethylmethanamide (DMF), under Ar atmosphere 2-Ethylhexyl bromide (5.79 g, 30 mmol) was added dropwise via a syringe. The mixture was heated to 100 ^o^C overnight in the dark. The reaction solvent was removed under vacuum and extracted with ethyl acetate and water. The combined organic phases were dried over magnesium sulfate. After filtration, the solvents were evaporated under vacuum and the crude product (compound 2) was purified on a silica gel using dichloromethane/hexane (1:1, v/v) as the eluent to give an orange-red solid (3.46 g, 90 %). ^1^H NMR (400 MHz, CDCl_3_) *δ* 7.09 (d, *J* = 0.6 Hz, 1H), 6.96 (d, *J* = 0.6 Hz, 1H), 4.40-4.25 (m, 4H), 1.86 (s, 1H), 1.36 (t, *J* = 7.1 Hz, 3H), 1.29-1.17 (m, 8H), 0.90-0.80 (m, 6H). ^13^C NMR (100 MHz, CDCl_3_) *δ* 160.84 (s), 142.65 (s), 125.21 (s), 115.00 (s), 113.49 (s), 108.54 (s), 59.54 (s), 50.96 (s), 39.97 (s), 29.78 (s), 27.76 (s), 23.13 (s), 22.41 (s), 13.79 (s), 13.35 (s), 10.05 (s). MS (MALDI-TOF) [M] calcd. for (C_17_H_24_BrNO_2_S):385.0708. Found: 385.0711.

**Synthesis of Compound 4:**

To a stirred solution of Compound **2** (3.85 g, 10 mmol) in a EtOH (50 mL) was added 1 M aqueous NaOH solution (50 mL). The mixture was refluxed overnight, and then cooled to room temperature. The organic volatiles were removed by rotary evaporation, and the residue was added 35% HCl slowly until reaching a pH of 1. The pale yellow precipitate was collected by filtration, washed with water and the air-dried to obtain compound **3**, which was used without further purification. Compound **3** (1.79g, 5mmol), silver acetate (835mg, 5mmol) and K_2_CO_3_ (690 mg, 5mmol) were added into a Schlenk flask. The flask was purged by argon for 3 times and then N-Methyl pyrrolidone (30ml) was added. The mixture was refluxed for 12h at 150^o^C. After reaction, the mixture was poured into ice water to cool and then extracted with ethyl acetate. The organic phase was collected and dried over anhydrous sodium sulfate. The solvent was removed under vacuum. The solvent was removed under vacuum and the crude product was purified by column chromatography over silica gel with hexane as the eluent to give compound **4**. It should be mentioned that compound **4** is not stable when the purity of compound **4** is high enough, therefore, when we purify compound **4**, we will not spin dry the eluent (hexane). So the NMR spectrum of compound **4** in CDCl_3_ contains peaks of hexane. ^1^H NMR (400 MHz, CDCl_3_) *δ* 6.94 (s, 1H), 6.82 (d, *J* = 2.9 Hz, 1H), 6.28 (d, *J* = 2.9 Hz, 1H), 3.86 (dd, *J* = 7.2, 4.5 Hz, 2H), 1.83 – 1.74 (m, 1H), 1.29-1.17 (m, 8H), 0.90-0.80 (m, 6H). ^13^C NMR (100 MHz, CDCl_3_) *δ* 124.82 (s), 122.39 (s), 112.95 (s), 108.72 (s), 99.35 (d, J = 1.9 Hz), 51.76 (s), 40.00 (s), 29.89 (s), 27.98 (s), 23.19 (s), 22.35 (s), 13.32 (s), 9.88 (s).

**Synthesis of Compound 5:**

To a stirring solution of Compound 4 (3.13 g, 10 mmol) in 40 mL of anhydrous THF was dropwise added n-BuLi (2.5 M in hexanes, 4.0 mL, 10.0 mmol) at −78°C under the protection of argon. The reaction mixture was stirred at−78°C for 1 h, followed by the addition of triisopropylsilyl chloride (2.5 mL, 11 mmol) in one portion, and then slowly warmed to room temperature. Finally, the reaction was quenched with water, and the mixture was extracted with hexanes. The combined extracts were washed with brine, dried over anhydrous Na_2_SO4, and then filtered. The solvent was removed by rotary evaporation to yield the crude product, which was then purified by column chromatography on silica gel with hexanes as the eluent to afford compound **5** (1.96 g, 50%). ^1^H NMR (400 MHz, CDCl_3_) *δ* 7.07 (s, 1H), 6.90 (d, *J* = 2.9 Hz, 1H), 6.42 (d, *J* = 2.8 Hz, 1H), 3.98 (d, *J* = 7.0 Hz, 2H), 1.88 (dd, *J* = 11.9, 5.9 Hz, 1H), 1.74-1.67 (m, 2H), 1.48-1.29 (m, 9H), 1.24-1.13 (m, 18H), 1.04 – 0.92 (m, 6H). ^13^C NMR (100 MHz, CDCl_3_) *δ*142.02 (s), 131.68 (s), 127.85 (s), 126.40 (s), 117.15 (s), 99.31 (s), 51.56 (s), 40.32 (s), 31.08 (s), 30.10 (s), 28.13 (s), 27.75 (s), 26.24 (s), 23.37 (s), 22.39 (s), 22.14 (s), 18.10 (s), 17.47 (s), 16.67 (s), 13.52 (d, *J* = 11.1 Hz), 13.04 (s), 11.39 (d, *J* = 18.4 Hz), 10.12 (s). MS (MALDI-TOF) [M] calcd. for (C_23_H_41_NSSi):391.2729. Found: 391.2792.

**Synthesis of compound 8:** **, and compound 9:**

To a stirring solution of compound **4** (3.13 g, 10.0 mmol) in 50 mL of anhydrous THF was dropwise added n-BuLi (2.5 M in hexanes, 4.0 mL,10.0 mmol) at −78°C under the protection of argon. The reaction mixture was stirred at −78°C for 1 h, followed by the addition of tributyltin chloride (3.0 mL, 11.0 mmol) in one portion, and then slowly warmed to room temperature. Finally, the reaction was quenched with water, and the mixture was extracted with hexanes. The combined extracts were washed with brine, dried over anhydrous Na_2_SO4, and then filtered. The solvent was removed by rotary evaporation to yield compound **7** as a yellow oil. The stannylated compound was used without further purification. To a two-neck flask were added compound **7** (5.22 g, 10 mmol), 2,5-dibromoterephthalic acid diethyl ester (3.42 g, 9.0 mmol), tributyl(thieno[3,2-*b*]thiophen-2-yl)stannane (4.30 g, 10.0 mmol), PdCl_2_(PPh_3_)_2_ (115 mg, 3 mol %), and anhydrous toluene (25 mL) under an argon atmosphere. The reaction mixture was then heated at 110°C for 1 day. The solvent was removed by rotary evaporation, and the crude product was purified by column chromatography on silica gel with hexanes and CH_2_Cl_2_ [1:1(v/v)] as the eluent to afford compound **8** (1.86 g, 30%) and compound **9** (1.71 g, 32%).

For compound **8**: ^1^H NMR (400 MHz, CDCl_3_) *δ* 7.85 (s, 2H), 7.02 (s, 2H), 6.87 (s, 2H), 6.40 (s, 2H), 4.28 (dd, *J* = 13.5, 6.6 Hz, 4H), 3.93 (s, 4H), 1.89 (s, 2H), 1.38-1.30 (m, 12H), 1.20-1.10 (m, 6H), 0.94 (s, 10H), 0.80-0.75 (m, 6H).^13^C NMR (100 MHz, CDCl_3_) *δ* 167.89 (s), 139.26 (s), 137.03 (s), 133.18 (d, *J* = 9.0 Hz), 130.59 (s), 125.90 (s), 123.44 (s), 109.66 (s), 99.51 (s), 61.02 (s), 51.76 (s), 40.02 (s), 29.92 (s), 28.05 (s), 23.24 (s), 22.42 (s), 13.50 (s), 13.29 (s), 10.04 (s). MS (MALDI-TOF) [M+Na] calcd. for (C_40_H_52_N_2_O_4_S_2_Na):711.3266. Found: 711.3268.

For compound **9**: ^1^H NMR (400 MHz, CDCl_3_) *δ* 7.91 (s, 1H), 7.80 (s, 1H), 7.33 (d, *J* = 5.2 Hz, 1H), 7.26 (s, 1H), 7.21 (d, *J* = 5.3 Hz, 1H), 6.99 (s, 1H), 6.81 (d, *J* = 2.9 Hz, 1H), 6.35 (d, *J* = 2.8 Hz, 1H), 4.22 (dd, *J* = 13.8, 6.9 Hz, 4H), 3.87 (dd, *J* = 7.0, 5.1 Hz, 2H), 1.82 (d, *J* = 8.3 Hz, 1H), 1.30-1.24 (m, 8H), 1.20-1.08 (m, 6H), 0.88 (t, *J* = 7.3 Hz, 6H). ^13^C NMR (100 MHz, CDCl_3_) *δ* 167.67 (s), 167.08 (s), 141.72 (s), 139.24 (d, *J* = 11.2 Hz), 138.77 (s), 136.75 (s), 134.16 (s), 133.47 (s), 133.25 (s), 132.12 (s), 131.03 (d, *J* = 7.4 Hz), 128.36 (s), 127.81 (s), 126.71 (s), 126.06 (s), 124.82 (s), 123.67 (s), 118.83 (s), 118.53 (s), 109.89 (s), 99.58 (s), 61.12 (s), 51.77 (s), 40.04 (s), 29.93 (s), 28.06 (s), 23.25 (s), 22.44 (s), 13.52 (s), 13.29 (d, *J* = 6.1 Hz), 10.05 (s). MS (MALDI-TOF) [M+Na] calcd. for (C_32_H_35_NO_4_S_3_Na):616.1626. Found: 616.1624.

**Synthesis of compound 10:**  **, and compound 11:**

To a stirring solution of compound **5** (3.91 g, 10.0 mmol) in 50 mL of anhydrous THF was dropwise added n-BuLi (2.5 M in hexanes, 4.0 mL,10.0 mmol) at 0°C under the protection of argon. The reaction mixture was stirred at 0°C for 1 h, followed by the addition of tributyltin chloride (3.0 mL, 11.0 mmol) in one portion, and then slowly warmed to room temperature. Finally, the reaction was quenched with water, and the mixture was extracted with hexanes. The combined extracts were washed with brine, dried over anhydrous Na_2_SO4, and then filtered. The solvent was removed by rotary evaporation to yield compound **6** as a yellow oil. The stannylated compound was used without further purification. To a two-neck flask were added compound **6** (6.81 g, 10 mmol), 2,5-dibromoterephthalic acid diethyl ester (3.42 g, 9.0 mmol), tributyl(thieno[3,2-*b*]thiophen-2-yl)stannane (4.30 g, 10.0 mmol), PdCl_2_(PPh_3_)_2_ (115 mg, 3 mol %), and anhydrous toluene (25 mL) under an argon atmosphere. The reaction mixture was then heated at 110°C for 1 day. Finally, the reaction was quenched with water, and the mixture was extracted with hexanes. The combined extracts were washed with brine, dried over anhydrous Na_2_SO4, and then filtered. The solvent was removed to obtain the crude product, then to the solution of the crude product in anhydrous THF was dropwise added TBAF (1 M in THF, 15.00 mL, 15.00 mmol) in the absence of light at 0°C. The reaction mixture was kept at 0°C for 30 min, and ethanol was then added. The solvent was removed by rotary evaporation, and the crude product was purified by column chromatography on silica gel with hexanes and CH_2_Cl_2_ [1:1(v/v)] as the eluent to afford compound **10** (1.49 g, 28%) and compound **11** (1.55 g, 25%).

For compound **10**: ^1^H NMR (400 MHz, CDCl_3_) *δ* 8.07 (s, 1H), 7.85 (s, 1H), 7.40 (d, *J* = 5.2 Hz, 1H), 7.33 (d, *J* = 0.4 Hz, 1H), 7.28 (dd, *J* = 5.2, 0.5 Hz, 1H), 7.10 (d, *J* = 5.2 Hz, 1H), 6.96 (d, *J* = 5.2 Hz, 1H), 6.41 (s, 1H), 4.26 (q, *J* = 7.1 Hz, 2H), 4.16 (qd, *J* = 7.1, 1.7 Hz, 2H), 3.83 (dd, *J* = 13.8, 7.3 Hz, 2H), 1.70-1.63 (m, 1H), 1.20 – 0.97 (m, 14H), 0.81 (t, *J* = 7.0 Hz, 3H), 0.72 (t, *J* = 7.4 Hz, 3H). ^13^C NMR (100 MHz, CDCl_3_) *δ* 166.82 (s), 166.25 (s), 141.53 (s), 140.78 (s), 139.29 (s), 138.76 (s), 135.27 (s), 133.80 (s), 133.49 (s), 133.15 (s), 132.65 (s), 132.14 (s), 131.90 (s), 126.77 (s), 122.55 (s), 122.17 (s), 118.76 (d, *J* = 12.4 Hz), 110.11 (s), 101.23 (s), 61.06 (d, *J* = 19.7 Hz), 52.87 (s), 49.34 (s), 39.56 (s), 29.79 (s), 27.78 (s), 23.19 (s), 22.20 (s), 17.12 (s), 13.46 (s), 13.21 (d, *J* = 7.3 Hz), 11.70 (s), 9.93 (s). MS (MALDI-TOF) [M+Na] calcd. for (C_32_H_35_NO_4_S_3_Na):616.1626. Found: 616.1629.

For compound **11**: ^1^H NMR (400 MHz, CDCl_3_) *δ* 8.03 (s, 2H), 7.09 (d, *J* = 5.2 Hz, 2H), 6.97 (d, *J* = 5.2 Hz, 2H), 6.44 (s, 2H), 4.18 (q, *J* = 7.1 Hz, 4H), 3.95 – 3.75 (m, 4H), 1.72 (d, *J* = 5.1 Hz, 2H), 1.25 – 0.99 (m, 22H), 0.83 (t, *J* = 7.0 Hz, 6H), 0.72 (t, *J* = 7.3 Hz, 6H).

^13^C NMR (100 MHz, CDCl_3_) *δ* 166.21 (s), 140.74 (s), 135.43 (s), 133.85 (s), 133.08 (s), 132.15 (s), 122.52 (s), 122.21 (s), 110.10 (s), 101.20 (s), 60.89 (s), 52.89 (s), 49.37 (s), 39.49 (s), 29.86 (s), 27.80 (s), 23.03 (s), 22.28 (s), 17.14 (s), 13.42 (s), 13.17 (s), 11.74 (s), 9.85 (s). MS (MALDI-TOF) [M+Na] calcd. for (C_40_H_52_N_2_O_4_S_2_Na):711.3266. Found: 711.3276.

**Synthesis of compound 12:**

To a solution of 4-hexyl-1-bromobenzene (930 mg, 3.9 mmmol) in THF (10 mL) at –78 ° C was added n-BuLi (1.54 mL, 3.9 mmol, 2.5 M in hexane) and the mixture was kept at –78 ° C for 1 h. A solution of compound **8** (550 mg, 0.80 mmol) in THF (10 mL) was then added slowly. After this addition, the mixture was stirred at room temperature overnight and then poured into water and extracted twice with ethyl acetate. The combined organic phase was dried over Na_2_SO_4_. After removing the solvent, the crude product was charged into three-neck flask. Acetic acid (20 mL) and concentrated H_2_SO_4_ (0.4 mL) were added and the mixture was refluxed for 3 h. Then the mixture was poured into water and extracted with ethyl acetate. The resulting crude compound was purified by silica gel column using a mixture of hexane/DCM as the eluent to give a light yellow solid (773 mg, 80%). ^1^H NMR (400 MHz, CDCl_3_) *δ* 7.38-7.30 (m, 10H), 7.10 (d, *J* = 8.1 Hz, 8H), 6.75 (d, *J* = 3.0 Hz, 2H), 6.46 (d, *J* = 3.0 Hz, 2H), 3.47 (d, *J* = 7.8 Hz, 4H), 2.64-2.56 (m, 8H), 1.62 (d, *J* = 5.6 Hz, 8H), 1.46-1.29 (m, 26H), 1.25-1.14 (m, 4H), 1.03-0.83 (m, 30H), 0.65 (t, *J* = 7.4 Hz, 6H). ^13^C NMR (100 MHz, CDCl_3_) *δ* 155.25 (s), 140.89 (s), 140.07 (s), 138.71 (d, *J* = 4.7 Hz), 135.03 (d, *J* = 13.0 Hz), 128.43 (s), 127.59 (s), 125.42 (s), 123.54 (s), 113.90 (s), 101.22 (s), 61.71 (s), 52.42 (s), 38.65 (s), 34.99 (s), 31.18 (s), 30.86 (d, *J* = 3.9 Hz), 29.26 (s), 28.56 (d, *J* = 4.4 Hz), 28.05 (s), 22.49 (d, *J* = 15.5 Hz), 22.05 (s), 13.53 (d, *J* = 5.6 Hz), 10.04 (s). MS (MALDI-TOF) [M] calcd. for (C_84_H_108_N_2_S_2_):1208.7954. Found: 1208.8027.

**Synthesis of compound 13:**

The synthetic route of compound **13** is similar to that of **12**, excepting for using compound **9** to replace compound **8**. The residue was purified by column chromatography on silica gel using hexane/DCM (8:1) as eluent, yielding a yellow solid (yield: 72%). ^1^H NMR (400 MHz, CDCl_3_) *δ* 7.57 (d, *J* = 2.7 Hz, 2H), 7.49 (dd, *J* = 8.3, 3.6 Hz, 4H), 7.37-7.32 (m, 5H), 7.30-7.26 (m, 1H), 7.22 (t, *J* = 7.1 Hz, 8H), 6.88 (d, *J* = 3.0 Hz, 1H), 6.57 (d, *J* = 3.0 Hz, 1H), 3.60 (d, *J* = 7.8 Hz, 2H), 2.71 (dd, *J* = 15.9, 8.8 Hz, 8H), 1.71 (d, *J* = 15.3 Hz, 8H), 1.48-1.38 (m, *J* = 8.9 Hz, 26H), 1.10-0.94 (m, 22H), 0.76 (t, *J* = 7.4 Hz, 3H). ^13^C NMR (100 MHz, CDCl_3_) *δ* 155.71 (s), 152.32 (s), 144.85 (s), 143.03 (s), 141.08 (d, *J* = 9.8 Hz), 140.62 (s), 140.03 (s), 139.79 (s), 139.35 (s), 138.60 (d, *J* = 6.4 Hz), 136.05 (s), 134.96 (s), 134.74 (s), 133.31 (s), 128.50 (s), 128.05-127.41 (m), 126.11 (s), 125.38 (s), 124.00 (s), 119.75 (s), 115.18 (d, *J* = 23.8 Hz), 101.43 (s), 62.36 (s), 61.90 (s), 52.89 (s), 52.54 (s), 38.74 (s), 35.09 (d, *J* = 4.5 Hz), 31.35-30.65 (m), 29.35 (s), 28.82 -28.50 (m), 28.13 (s), 22.59 (d, *J* = 16.3 Hz), 22.16 (d, *J* = 8.0 Hz), 13.61 (d, *J* = 4.2 Hz), 10.11 (s). MS (MALDI-TOF) [M+Na] calcd. for (C_76_H_91_NS_3_):1136.6211. Found: 1136.6245.

**Synthesis of compound 14:**

The synthetic route of compound **14** is similar to that of **12**, excepting for using compound **10** to replace compound **8**. The residue was purified by column chromatography on silica gel using hexane/DCM (8:1) as eluent, yielding a yellow solid (yield: 68%). ^1^H NMR (400 MHz, CDCl_3_) *δ* 7.67 (s, 1H), 7.55 (s, 1H), 7.38 (dd, *J* = 8.1, 1.8 Hz, 4H), 7.35 – 7.30 (m, 5H), 7.28 (d, *J* = 5.2 Hz, 1H), 7.20 (dd, *J* = 8.0, 4.0 Hz, 8H), 6.94 (q, *J* = 5.2 Hz, 2H), 4.07 (d, *J* = 7.4 Hz, 2H), 2.68 (t, *J* = 7.7 Hz, 8H), 1.97 (d, *J* = 5.4 Hz, 1H), 1.69 (t, *J* = 10.2 Hz, 8H), 1.50-1.18 (m, 32H), 1.05-0.96 (m, 12H), 0.92 (t, *J* = 7.4 Hz, 3H), 0.85 (t, *J* = 6.5 Hz, 3H). ^13^C NMR (100 MHz, CDCl_3_) *δ* 152.60 (s), 152.09 (s), 144.72 (s), 143.22 (s), 141.91 (d, *J* = 3.5 Hz), 141.09 (s), 140.66 (d, *J* = 19.2 Hz), 140.13 (s), 139.39 (s), 133.69 (s), 133.27 (s), 132.50 (s), 128.49 (s), 127.83 (d, *J* = 8.9 Hz), 127.44 (d, *J* = 17.1 Hz), 125.35 (s), 121.80 (s), 119.86 (s), 117.47 (s), 114.83 (s), 110.45 (s), 62.45 (s), 58.95 (s), 50.53 (s), 40.28 (s), 35.12 (s), 31.23 (d, *J* = 1.9 Hz), 30.83 (d, *J* = 2.1 Hz), 29.99 (s), 28.85-28.47 (m), 28.16 (s), 23.45 (s), 22.44 (s), 22.11 (d, *J* = 1.2 Hz), 13.62 (d, *J* = 1.3 Hz), 13.40 (s), 10.17 (s). MS (MALDI-TOF) [M] calcd. for (C_76_H_91_NS_3_):1113.6314. Found: 1113.6351.

**Synthesis of compound 15:**

The synthetic route of compound **15** is similar to that of **12**, excepting for using compound **11** to replace compound **8**. The residue was purified by column chromatography on silica gel using hexane/DCM (8:1) as eluent, yielding a yellow solid (yield: 35%). ^1^H NMR (400 MHz, CDCl_3_) *δ* 7.48 (s, 2H), 7.23 (dd, *J* = 8.3, 2.1 Hz, 8H), 7.06 (d, *J* = 8.0 Hz, 8H), 6.96 (d, *J* = 5.2 Hz, 2H), 6.92 (d, *J* = 5.3 Hz, 2H), 4.11 (d, *J* = 7.5 Hz, 4H), 2.58-2.53 (m, 8H), 1.93 (d, *J* = 6.1 Hz, 2H), 1.65-1.57 (m, 6H), 1.42-1.23 (m, 36H), 1.21-1.15 (m, 6H), 0.98-0.80 (m, 18H), 0.77 (t, *J* = 6.9 Hz, 6H). ^13^C NMR (100 MHz, CDCl_3_) *δ* 151.87 (s), 144.23 (s), 141.97 (s), 140.34 (s), 139.77 (s), 130.52 (s), 127.60 (s), 127.15 (s), 121.09 (s), 117.43 (s), 115.83 (s), 110.37 (s), 58.82 (s), 50.67 (s), 40.21 (s), 34.99 (s), 31.13 (s), 30.76 (s), 29.88 (s), 28.57 (d, *J* = 3.4 Hz), 27.96 (s), 23.33 (s), 22.37 (s), 22.00 (s), 13.50 (s), 13.29 (s), 10.05 (s), 0.55 (s). MS (MALDI-TOF) [M] calcd. for (C_84_H_108_N_2_S_2_):1208.7954. Found: 1208.8022.

**Synthesis of compound 16:**

To a dry 100 mL two-necked round bottom flask, 10 ml anhydrous N, N-dimethylformamide (DMF) was added, and the solution was cooled to 0 ^o^C and stirred when 2 ml phosphorous oxychloride (POCl_3_) was added by syringe under argon protection. The mixture kept at 0 ^o^C for 2h, and then compound **12** (604 mg, 0.5 mmol) in dry 1, 2-dichloroethane (20 ml) was added. Then, the mixture solution was allowed to reflux overnight. After cooling to room temperature, 100 ml water was added to quench the reaction. The mixture was extracted with dichloromethane, and the organic layer was collected, washed with water and dried with anhydrous Na_2_SO_4_. After removal of the solvent under reduced pressure, the residue was purified by column chromatography on silica gel using a mixture solvent as eluent (hexane/dichloromethane, v/v = 1/1) to give a yellow solid (538 mg, 85%). ^1^H NMR (400 MHz, CDCl_3_) *δ* 9.52 (s, 2H), 7.39 (s, 2H), 7.27 (dd, *J* = 8.1, 2.1 Hz, 8H), 7.20 (s, 2H), 7.11 (d, *J* = 7.2 Hz, 8H), 3.95 (d, *J* = 7.2 Hz, 4H), 2.62-2.55 (m, 8H), 1.59 (dd, *J* = 14.7, 7.3 Hz, 8H), 1.41-1.27 (m, 30H), 1.15-0.90 (m, 16H), 0.82-0.75 (m, 6H), 0.54 (t, *J* = 6.9 Hz, 6H), 0.50-0.35 (m, 8H). ^13^C NMR (100 MHz, CDCl_3_) *δ* 178.40 (s), 157.04 (s), 149.05 (s), 142.91 (s), 141.54 (d, *J* = 3.9 Hz), 137.80 (s), 137.28 (s), 135.31 (s), 135.00 (s), 128.31 (s), 127.95 (d, *J* = 3.2 Hz), 126.27 (s), 117.43 (s), 115.02 (s), 62.26 (s), 52.20 (s), 39.64 (s), 34.94 (s), 31.11 (s), 30.83 (d, *J* = 4.3 Hz), 28.88 (s), 28.68-28.34 (m), 22.35 (d, *J* = 15.7 Hz), 21.98 (s), 13.44 (d, *J* = 11.2 Hz), 10.76 (s). MS (MALDI-TOF) [M] calcd. for (C_86_H_108_N_2_O_2_S_2_):1264.7852. Found: 1264.8071.

**Synthesis of compound 17:**

The synthetic route of compound **17** is similar to that of **16**, excepting for using compound **13** to replace compound **12**. The residue was purified by column chromatography on silica gel using hexane/DCM (1:1) as eluent, yielding a yellow solid (yield: 82%). ^1^H NMR (400 MHz, CDCl_3_) *δ* 9.87 (s, 1H), 9.61 (s, 1H), 7.89 (s, 1H), 7.63 (d, *J* = 1.9 Hz, 2H), 7.41 (d, *J* = 8.3 Hz, 4H), 7.26-7.15 (m, 13H), 4.07 (d, *J* = 7.0 Hz, 2H), 2.65 (dt, *J* = 13.4, 7.9 Hz, 8H), 1.65 (dd, *J* = 14.0, 7.0 Hz, 8H), 1.50-1.31 (m, 26H), 1.16-0.81 (m, 19H), 0.64 (t, *J* = 7.1 Hz, 3H), 0.51 (dd, *J* = 30.1, 5.9 Hz, 3H). ^13^C NMR (100 MHz, CDCl_3_) *δ* 182.15 (s), 178.55 (s), 157.54 (s), 153.62 (s), 149.25 (s), 148.76 (s), 145.59 (s), 143.54 (s), 142.82 (s), 141.88 -141.52 (m), 140.88 (s), 139.64 (s), 138.81 (s), 138.38 (s), 137.19 (s), 135.82 (s), 135.50 (s), 135.24 (s), 129.29 (s), 128.39 (s), 128.12 (s), 127.29 (s), 126.68 (s), 117.49 (s), 116.43 (s), 116.14 (s), 62.44 (d, *J* = 3.1 Hz), 52.89 (s), 52.30 (s), 39.69 (s), 35.01 (s), 31.15 (d, *J* = 2.0 Hz), 31.01-30.57 (m), 28.98 (s), 28.75-28.25 (m), 22.44 (d, *J* = 15.9 Hz), 22.09 (d, *J* = 9.5 Hz), 13.52 (d, *J* = 9.7 Hz), 10.84 (s). MS (MALDI-TOF) [M] calcd. for (C_78_H_91_NO_2_S_3_):1169.6212. Found: 1169. 6240.

**Synthesis of compound 18:**

The synthetic route of compound **18** is similar to that of **16**, excepting for using compound **14** to replace compound **12**. The residue was purified by column chromatography on silica gel using hexane/DCM (1:1) as eluent, yielding a yellow solid (yield: 88%). ^1^H NMR (400 MHz, CDCl_3_) *δ* 9.79 (s, 1H), 9.71 (s, 1H), 7.81 (s, 1H), 7.67 (s, 1H), 7.58 (s, 1H), 7.43 (s, 1H), 7.21 (dd, *J* = 8.2, 2.7 Hz, 4H), 7.15 (dd, *J* = 8.3, 2.6 Hz, 4H), 7.10 (dd, *J* = 7.1, 4.9 Hz, 8H), 4.16 (d, *J* = 7.5 Hz, 2H), 2.59-2.52 (m, 8H), 1.88 (d, *J* = 6.0 Hz, 1H), 1.62-1.67 (m, 8H), 1.37-1.07 (m, 32H), 0.95-0.83 (m, 15H), 0.73 (t, *J* = 6.9 Hz, 3H). ^13^C NMR (100 MHz, CDCl_3_) *δ* 182.13 (d, *J* = 22.9 Hz), 182.01-181.86 (m), 154.30 (s), 153.59 (s), 149.34 (s), 145.55 (s), 145.01 (s), 144.52 (s), 143.53 (s), 141.70 (s), 141.12 (d, *J* = 12.9 Hz), 140.61 (d, *J* = 4.2 Hz), 139.60 (d, *J* = 8.1 Hz), 138.83 (s), 134.63 (s), 132.02 (s), 129.43 (s), 129.19 (s), 128.08 (d, *J* = 12.3 Hz), 127.12 (d, *J* = 12.8 Hz), 125.22 (s), 118.54 (s), 116.25 (s), 62.55 (s), 58.94 (s), 50.85 (s), 40.23 (s), 35.01 (s), 31.14 (d, *J* = 1.1 Hz), 30.75 (s), 29.94 (s), 29.15 (s), 28.68-28.33 (m), 28.03 (s), 23.45 (s), 22.31 (s), 22.03 (s), 13.54 (s), 13.28 (s), 10.08 (s). MS (MALDI-TOF) [M+Na] calcd. for (C_78_H_91_NO_2_S_3_Na):1192.6110. Found: 1192. 6127.

**Synthesis of compound 19:**

The synthetic route of compound **19** is similar to that of **16**, excepting for using compound **15** to replace compound **12**. The residue was purified by column chromatography on silica gel using hexane/DCM (1:1) as eluent, yielding a yellow solid (yield: 85%). ^1^H NMR (400 MHz, CDCl_3_) *δ* 9.79 (s, 2H), 7.61 (s, 2H), 7.54 (s, 2H), 7.17 (dd, *J* = 8.2, 2.1 Hz, 8H), 7.08 (d, *J* = 7.9 Hz, 8H), 4.18 (d, *J* = 7.6 Hz, 4H), 2.56 (t, *J* = 7.7 Hz, 8H), 1.94 (s, 4H), 1.60-1.52 (m, 8H), 1.37-1.08 (m, 40H), 0.91-0.86 (m, 16H), 0.75 (t, *J* = 6.7 Hz, 6H). ^13^C NMR (100 MHz, CDCl_3_) *δ* 181.95 (s), 153.85 (s), 145.33 (s), 144.37 (s), 141.09 (s), 140.64 (s), 139.30 (s), 130.65 (s), 128.70 (s), 127.91 (s), 126.86 (s), 125.33 (s), 117.27 (s), 58.87 (s), 50.87 (s), 40.21 (s), 34.94 (s), 33.22 (s), 31.32 (s), 31.08 (s), 30.72 (s), 29.88 (s), 29.57 (s), 29.24-28.71 (m), 28.45 (t, *J* = 9.1 Hz), 27.93 (s), 23.37 (s), 22.29 (s), 22.03 (d, *J* = 12.3 Hz), 13.50 (d, *J* = 5.1 Hz), 13.22 (s), 10.01 (s). MS (MALDI-TOF) [M] calcd. for (C_86_H_108_N_2_O_2_S_2_):1264.7852. Found: 1264.7964.

**Synthesis of ThPy1:**

To a 100 ml round bottom flask, compound **16** (127 mg, 0.10 mmol), 2-(5,6-dichloro-3-oxo-2,3-dihydro-1H-inden-1-ylidene)malononitrile (131 mg, 0.5 mmol), chloroform (20 ml) were added under argon protection and stirred for a while when pyridine (1 ml) was added. The mixture was kept stirring at 70 ^o^C overnight. After removal of chloroform of reaction mixture under reduced pressure, 80 ml methanol was added and the precipitate was collected by filtration. The residue was purified by column chromatography on silica gel using a mixture solvent as eluent (hexane/dichloromethane, v/v = 2/3) to give a blue-dark solid (140 mg, 80%). ^1^H NMR (400 MHz, CDCl_3_) *δ* 9.03 (s, 2H), 8.70 (s, 2H), 8.40 (s, 2H), 7.86 (s, 2H), 7.40 (s, 2H), 7.24-7.19 (m, 8H), 7.12 (d, *J* = 8.2 Hz, 8H), 3.85 (d, *J* = 7.8 Hz, 4H), 2.57 (t, *J* = 7.6 Hz, 8H), 1.58-1.50 (m, 12H), 1.35-1.20 (m, 24H), 1.00-0.83 (m, 18H), 0.72-0.66 (m, 8H), 0.53-0.27 (m, 12H).^13^C NMR (100 MHz, CDCl_3_) *δ* 184.35 (s), 159.58 (s), 158.49 (s), 156.20 (s), 146.86 (s), 142.19 (d, *J* = 4.3 Hz), 138.16-137.67 (m), 137.37 (s), 136.18 (d, *J* = 8.4 Hz), 135.82 (s), 134.95 (s), 132.48 (s), 129.23 (s), 128.31 (d, *J* = 5.0 Hz), 128.07 (s), 125.76 (s), 124.03 (s), 121.67 (s), 117.97 (s), 116.00 (s), 114.96 (s), 114.53 (s), 65.25 (s), 62.27 (s), 51.33 (s), 41.49 (s), 34.91 (s), 31.06 (s), 30.75 (s), 29.31 (s), 28.87-28.46 (m), 22.96 (s), 22.22 (s), 21.95 (s), 13.46 (s), 13.25 (s), 11.05 (s). MS (MALDI-TOF) [M] calcd. for (C_110_H_112_Cl_4_N_6_O_2_S_2_):1755.7046. Found: 1755.7166.

**Synthesis of ThPy2:**

The synthetic route of **ThPy2** is similar to that of **ThPy1**, excepting for using compound **17** to replace compound **16**. The residue was purified by column chromatography on silica gel using hexane/DCM (1:2) as eluent, yielding a blue-dark solid (yield: 78%). ^1^H NMR (400 MHz, CDCl_3_) *δ* 9.05 (s, 1H), 8.85 (s, 1H), 8.75 (s, 1H), 8.71 (s, 1H), 8.42 (s, 1H), 8.21 (s, 1H), 7.94 (s, 1H), 7.88 (s, 1H), 7.53 (d, *J* = 9.4 Hz, 2H), 7.27 (s, 2H), 7.26-7.23 (m, 2H), 7.19-7.09 (m, 12H), 3.87 (d, *J* = 7.5 Hz, 2H), 2.57 (dd, *J* = 16.4, 8.4 Hz, 8H), 1.61-1.52 (m, 14H), 1.35-1.28 (m, 22H), 1.02-0.67 (m, 15H), 0.50-0.40 (m, 8H). ^13^C NMR (100 MHz, CDCl_3_) *δ* 185.18 (s), 184.34 (s), 159.55 (s), 158.83 (s), 157.41 (s), 156.12 (s), 154.94 (s), 153.44 (s), 147.39 (s), 146.93 (d, *J* = 16.5 Hz), 143.35 (s), 142.55-141.92 (m), 139.10 (d, *J* = 7.4 Hz), 138.82 (s), 138.54-137.35 (m), 136.95 (s), 136.70 (s), 136.20 (d, *J* = 7.4 Hz), 135.88 (d, *J* = 13.3 Hz), 135.30 (s), 134.98 (s), 132.60 (s), 129.29 (s), 128.27 (dd, *J* = 15.9, 9.6 Hz), 127.20 (s), 126.31 (s), 125.77 (s), 124.56 (s), 124.06 (s), 121.69 (s), 121.01 (s), 118.07 (s), 117.08 (d, *J* = 11.7 Hz), 114.95 (s), 114.50 (s), 113.58 (s), 69.23 (s), 65.38 (s), 62.46 (d, *J* = 11.1 Hz), 51.38 (s), 41.52 (s), 34.95 (d, *J* = 4.8 Hz), 31.07 (s), 30.69 (d, *J* = 11.2 Hz), 29.36 (s), 29.21 (d, *J* = 26.1 Hz), 28.63 (d, *J* = 14.6 Hz), 22.99 (s), 22.24 (s), 21.97 (s), 13.47 (s), 13.27 (s), 11.07 (s). MS (MALDI-TOF) [M] calcd. for (C_102_H_95_Cl_4_N_5_O_2_S_3_):1660.5406. Found: 1660.5557.

**Synthesis of ThPy3:**

The synthetic route of **ThPy3** is similar to that of **ThPy1**, excepting for using compound **18** to replace compound **16**. The residue was purified by column chromatography on silica gel using hexane/DCM (1:2) as eluent, yielding a blue-dark solid (yield: 84%). ^1^H NMR (400 MHz, CDCl_3_) *δ* 8.91-8.74 (m, 3H), 8.70 (s, 1H), 8.24 (s, 1H), 7.94 (s, 1H), 7.85 (s, 1H), 7.70 (s, 1H), 7.64 (s, 1H), 7.22-7.06 (m, 17H), 4.21 (s, 2H), 2.57 (t, *J* = 7.5 Hz, 8H), 1.92 (s, 1H), 1.58 (s, 12H), 1.42-1.07 (m, 31H), 0.90-0.78 (m, 14H), 0.74 (d, *J* = 6.3 Hz, 3H). ^13^C NMR (100 MHz, CDCl_3_) *δ* 185.25 (s), 157.41 (s), 156.54 (s), 155.06 (s), 147.17 (s), 142.08 (s), 141.59 (s), 139.62 (d, *J* = 4.1 Hz), 139.18 (d, *J* = 5.1 Hz), 138.48 (ddd, *J* = 28.1, 23.4, 5.5 Hz), 136.97 (s), 136.23 (s), 135.34 (d, *J* = 5.1 Hz), 131.28 (s), 131.07 (s), 128.24 (d, *J* = 11.3 Hz), 127.01 (d, *J* = 9.5 Hz), 126.35 (s), 125.99 (s), 124.58 (s), 124.00 (s), 121.20 (s), 119.23 (s), 117.49 (s), 114.49 (d, *J* = 19.3 Hz), 113.55 (s), 69.40 (s), 65.97 (s), 62.62 (s), 58.93 (s), 51.13 (s), 39.96 (s), 34.96 (s), 31.07 (d, *J* = 2.0 Hz), 30.68 (s), 29.76 (s), 29.08 (s), 28.51 (d, *J* = 4.7 Hz), 27.84 (s), 23.38 (s), 22.20 (s), 21.96 (s), 13.47 (d, *J* = 1.6 Hz), 13.19 (s), 10.01 (s). MS (MALDI-TOF) [M] calcd. for (C_102_H_95_Cl_4_N_5_O_2_S_3_):1660.5406. Found: 1660.5566.

**Synthesis of ThPy4:**

The synthetic route of **ThPy4** is similar to that of **ThPy1**, excepting for using compound **19** to replace compound **16**. The residue was purified by column chromatography on silica gel using hexane/DCM (1:2) as eluent, yielding a blue-dark solid (yield: 82%). ^1^H NMR (400 MHz, CDCl_3_) *δ* 8.81 (s, 2H), 8.70 (s, 2H), 7.85 (s, 2H), 7.67 (s, 2H), 7.14 (dd, *J* = 23.7, 7.6 Hz, 18H), 4.21 (s, 4H), 2.56 (t, *J* = 7.4 Hz, 8H), 1.94 (s, 2H), 1.59-1.55 (m, 12H), 1.42-1.09 (m, 40H), 0.92-0.83 (m, 14H), 0.75 (d, *J* = 6.1 Hz, 6H). ^13^C NMR (100 MHz, CDCl_3_) *δ* 185.33 (s), 157.95 (s), 156.31 (s), 150.80 (s), 141.64 (s), 139.58 (d, *J* = 3.5 Hz), 139.07-137.88 (m), 136.77 (s), 135.37 (s), 131.01 (d, *J* = 11.7 Hz), 128.18 (s), 126.81 (s), 126.00 (s), 124.01 (s), 118.58 (s), 114.47 (d, *J* = 19.2 Hz), 66.06 (s), 58.93 (s), 51.17 (s), 40.02 (s), 34.93 (s), 31.07 (s), 30.71 (s), 29.76 (s), 28.49 (d, *J* = 2.8 Hz), 27.79 (s), 23.36 (s), 22.23 (s), 21.96 (s), 13.46 (s), 13.19 (s), 10.01 (s). MS (MALDI-TOF) [M] calcd. for (C_110_H_112_Cl_4_N_6_O_2_S_2_):1755.7046. Found: 1755.7201.

**Table S1.** Optical and electrochemical properties of ThPy1, ThPy2, IT-4Cl, ThPy3 and ThPy4.

| Acceptor | λ_max_^sol^  (nm) | λ_max_^film^  (nm) | λ_onset_^film^  (nm) | *E*_g_^opt^*^a^*  (eV) | LUMO^CV^  (eV) | HOMO^CV^  (eV) |
| --- | --- | --- | --- | --- | --- | --- |
| ThPy1 | 744 | 772 | 849 | 1.46 | -3.87 | -5.59 |
| ThPy2 | 727 | 765 | 843 | 1.47 | -4.01 | -5.71 |
| IT-4Cl | 704 | 752 | 832 | 1.49 | -4.14 | -5.79 |
| ThPy3 | 749 | 819 | 919 | 1.35 | -4.04 | -5.64 |
| ThPy4 | 786 | 867 | 975 | 1.27 | -3.95 | -5.50 |

*^a^*Calculated from *E*_g_^opt^ =1240/*λ*_onset_.

**Table S2.** The parameters of exciton dissociation efficiency and charge collection efficiency.

| **Active layer** | ***J*_sat_ (mA/cm^2^)** | ***J*_ph_^a^ (mA/cm^2^)** | ***J*_ph_^b^ (mA/cm^2^)** | ***η*_diss_ (%)** | ***η*_coll_ (%)** |
| --- | --- | --- | --- | --- | --- |
| PM6:ThPy1 | 19.29 | 17.68 | 14.85 | 91.6 | 77.0 |
| PM6:ThPy2 | 21.32 | 19.63 | 16.86 | 92.1 | 79.1 |
| PM6:IT-4Cl | 22.44 | 20.89 | 18.53 | 93.1 | 82.6 |
| PM6:ThPy3 | 25.58 | 23.82 | 21.75 | 93.2 | 85.0 |
| PM6:ThPy4 | 18.38 | 16.70 | 13.58 | 90.8 | 73.9 |

a: Under short circuit condition; b: Under the maximal power output condition


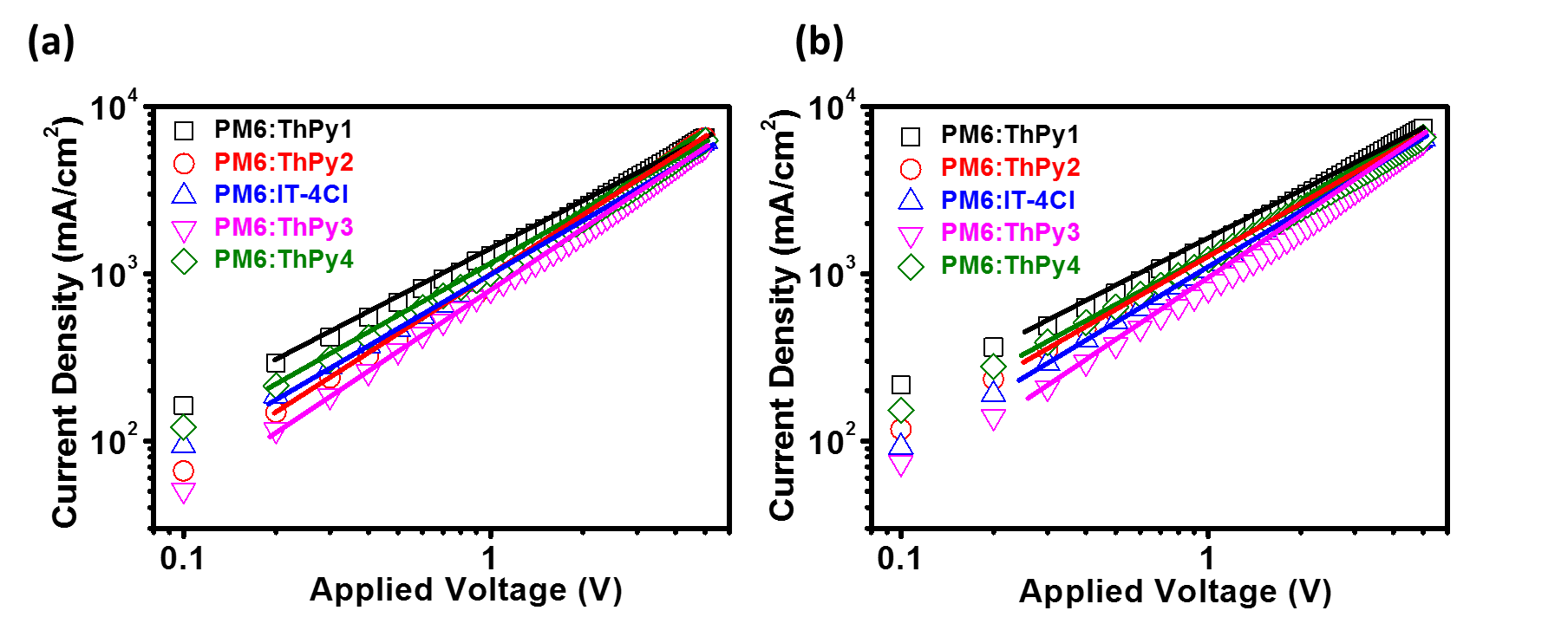


**Figure S1.** *J-V* characteristics in dark for hole-only (a) and electron-only (b) devices based on PM6:ThPy1, PM6:ThPy2, PM6:IT-4Cl, PM6:ThPy3 and PM6:ThPy4 blends.

**Table S3.** The parameters of hole mobilities and electron mobilities.

| Active layer | *μ*_h_  (10^-4^ cm^2^ V^-1^ s^-1^) | *μ_e_*  (10^-4^ cm^2^ V^-1^ s^-1^) | *μ_h_/μ_e_* |
| --- | --- | --- | --- |
| PM6:ThPy1 | 6.55 | 3.45 | 1.90 |
| PM6:ThPy2 | 8.59 | 4.82 | 1.78 |
| PM6:IT-4Cl | 8.23 | 5.05 | 1.63 |
| PM6:ThPy3 | 8.82 | 5.51 | 1.60 |
| PM6:ThPy4 | 7.59 | 3.89 | 1.95 |

**Table S4**. Summary of parameters measured and calculated from FTPS-EQE and EL.

| Device | *E*_g_^pv^  (eV) | EQE_EL_  10^-5^ | *E*_loss_  (eV) | *q*∆*V*_OC_^SQ^ (eV) | ∆*E*_1_=*E*_gap_-*q*∆*V*_OC_^SQ^  (eV) | *qV*_OC_^rad^  (eV) | ∆*E*_2_ =*q* ∆*V*_OC_^rad;below gap^  (eV) | ∆*E*_3_=*q*∆*V*_OC_^non-rad^ (eV) | *V*_OC_^cal^  (eV) |
| --- | --- | --- | --- | --- | --- | --- | --- | --- | --- |
| ThPy1 | 1.52 | 1.13 | 0.61 | 1.21 | 0.27 | 1.21 | 0.04 | 0.30 | 0.91 |
| ThPy2 | 1.51 | 0.315 | 0.66 | 1.18 | 0.27 | 1.18 | 0.06 | 0.33 | 0.85 |
| IT-4Cl | 1.54 | 0.029 | 0.74 | 1.19 | 0.27 | 1.19 | 0.08 | 0.39 | 0.80 |
| ThPy3 | 1.42 | 1.39 | 0.60 | 1.11 | 0.26 | 1.11 | 0.05 | 0.29 | 0.82 |
| ThPy4 | 1.34 | 12.4 | 0.51 | 1.06 | 0.26 | 1.06 | 0.02 | 0.23 | 0.83 |


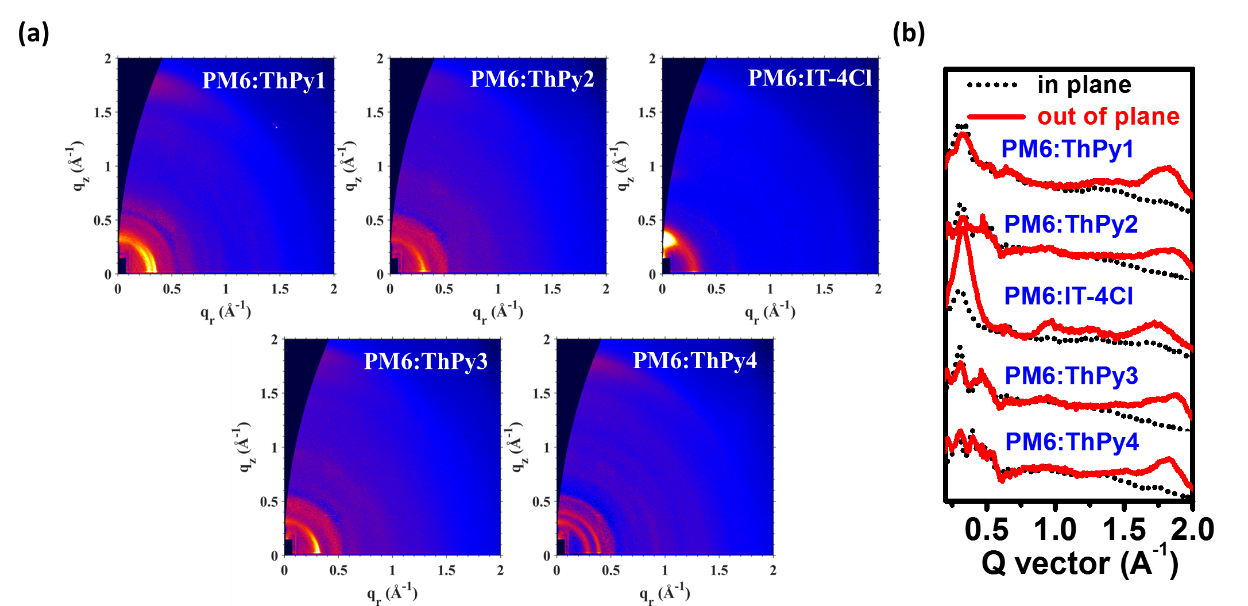


**Figure S2.** (a) GIWAXS patterns of PM6:ThPy1, PM6:ThPy2, PM6:IT-4Cl, PM6:ThPy3 and PM6:ThPy4 blend films; (b) Corresponding intensity profiles of PM6:ThPy1, PM6:ThPy2, PM6:IT-4Cl, PM6:ThPy3 and PM6:ThPy4 blend films along the in-plane (in black) and out-of-plane (in red) direction.


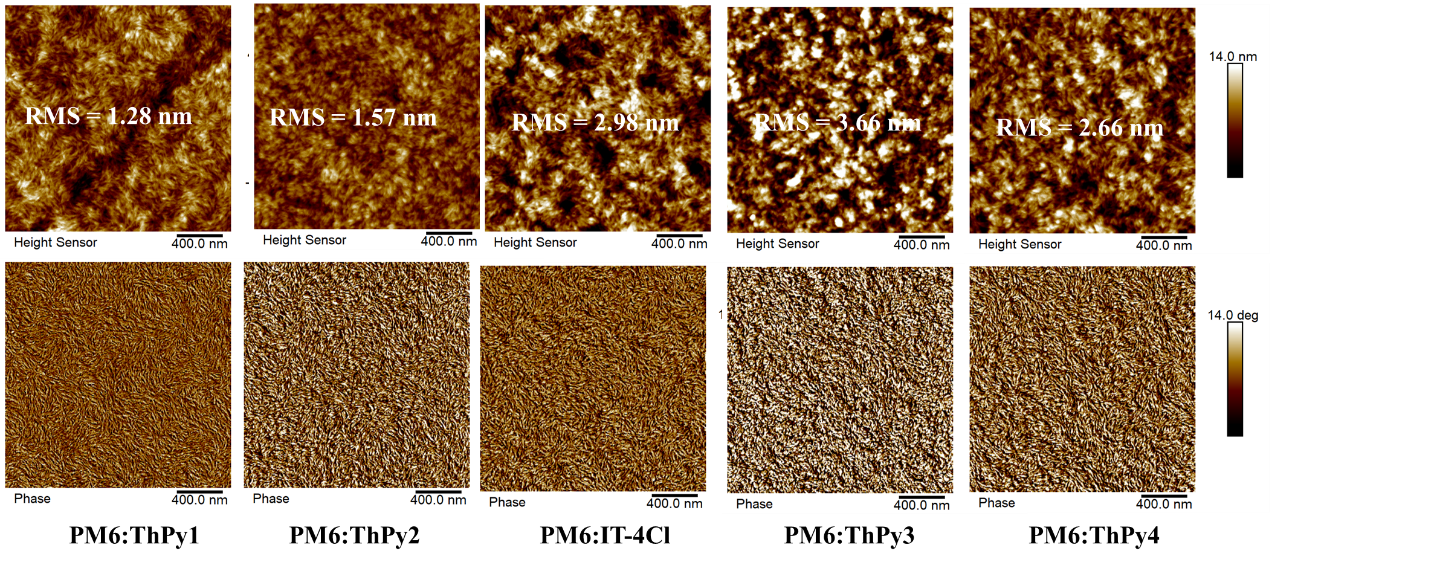


**Figure S3.** Atomic force microscopy (AFM) height (upper) and phase (lower) images of PM6:ThPy1, PM6:ThPy2, PM6:IT-4Cl, PM6:ThPy3 and PM6:ThPy4 blend films.

**Table S5**. The optimized photovoltaic performances of the OSCs based on PM6:BTP-eC9:acceptor (1:1.1:0.1) and PM6:BTP-eC9 under standard AM 1.5G illumination, 100 mW/cm^2^. The average values and standard deviations were obtained from 25 devices.

| Device | ***V*_OC_**  **(V)** | ***J*_SC_**  **(mA/cm^2^)** | **FF** | **PCE_max_**  **%** |
| --- | --- | --- | --- | --- |
| PM6:BTP-eC9:ThPy1 | 0.852 | 25.74 | 0.771 | 16.91 |
| PM6:BTP-eC9:ThPy2 | 0.848 | 26.11 | 0.761 | 16.84 |
| PM6:BTP-eC9:ThPy3 | 0.846 | 25.96 | 0.788 | 17.31 |
| PM6:BTP-eC9:ThPy4 | 0.849 | 27.08 | 0.802 | 18.43 |
| PM6:BTP-eC9 | 0.847 | 26.41 | 0.783 | 17.51 |


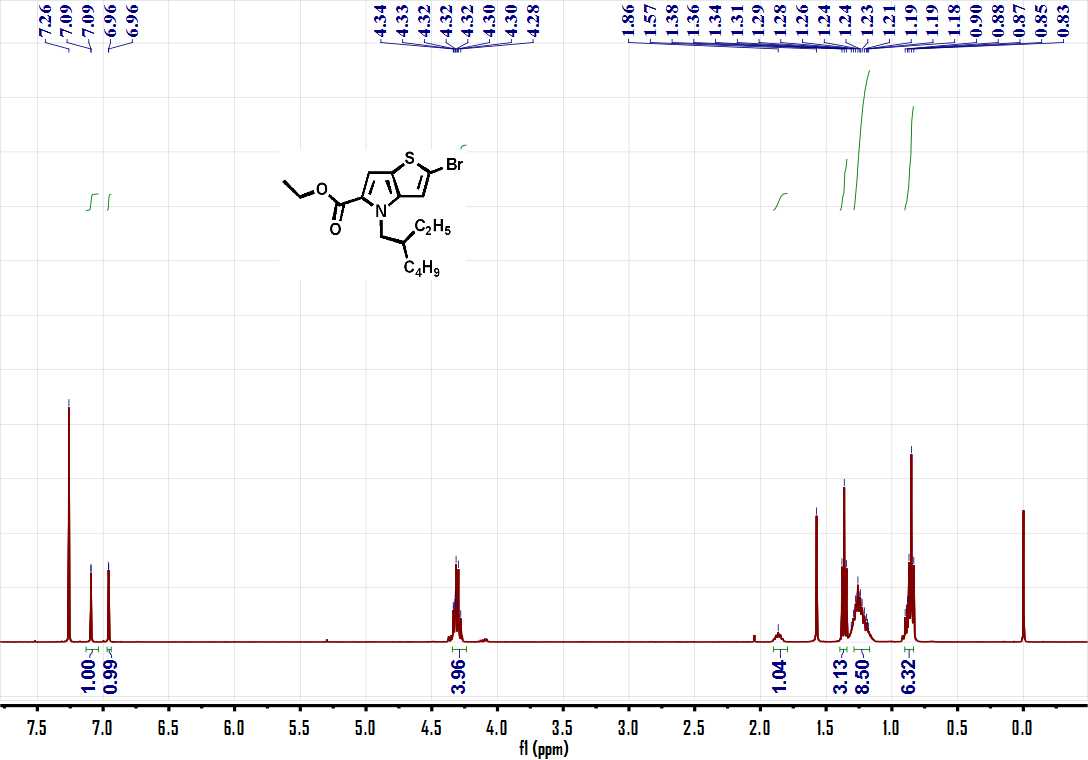


**Figure S4**. ^1^H NMR spectrum of Compound **2** in CDCl_3_.


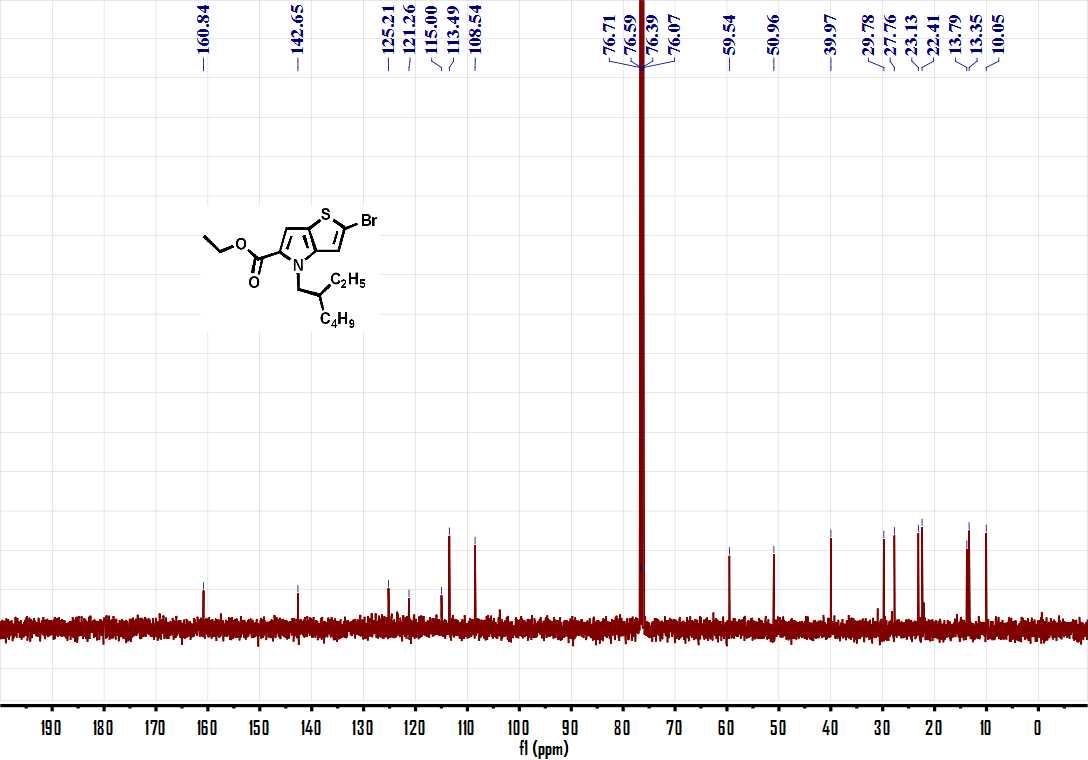


**Figure S5**. ^13^C NMR spectrum of Compound **2** in CDCl_3_.


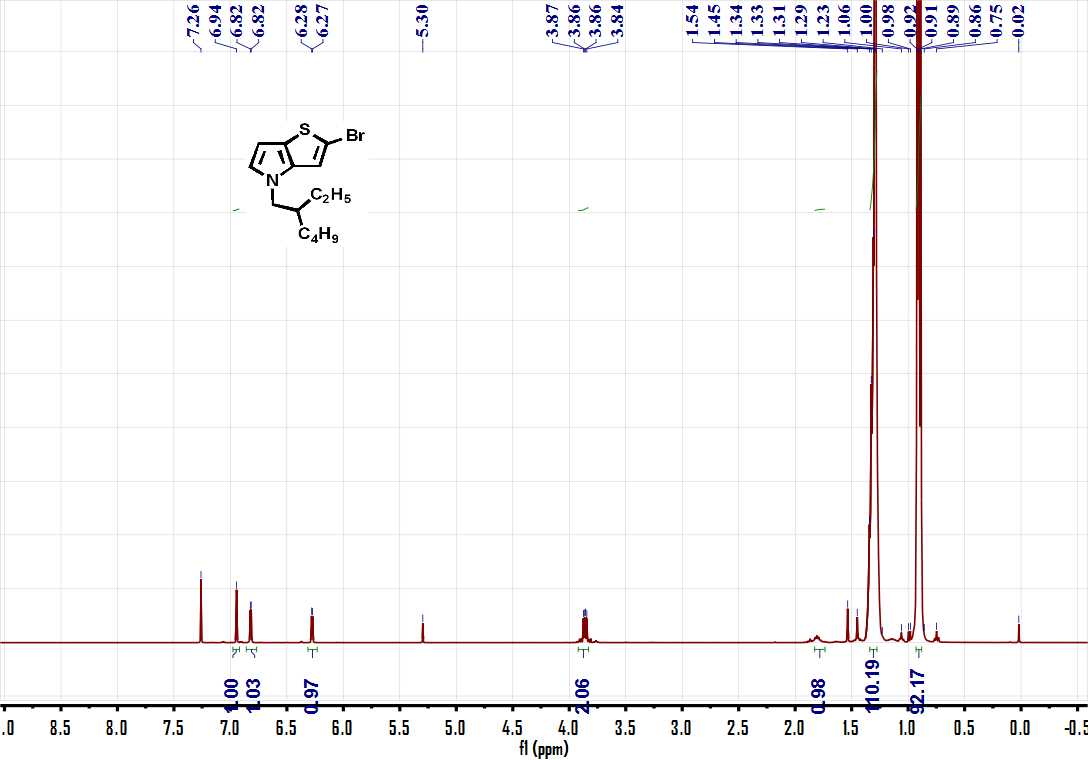


**Figure S6**. ^1^H NMR spectrum of Compound **4** in CDCl_3_.


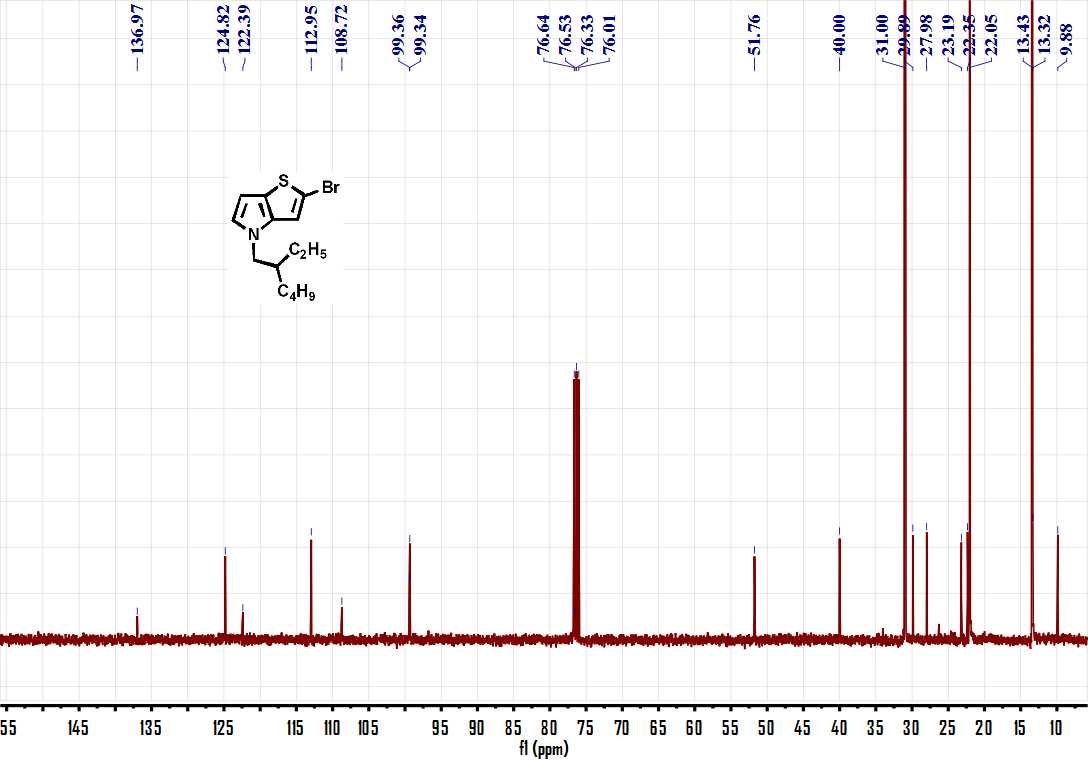


**Figure S7**. ^13^C NMR spectrum of Compound **4** in CDCl_3_.


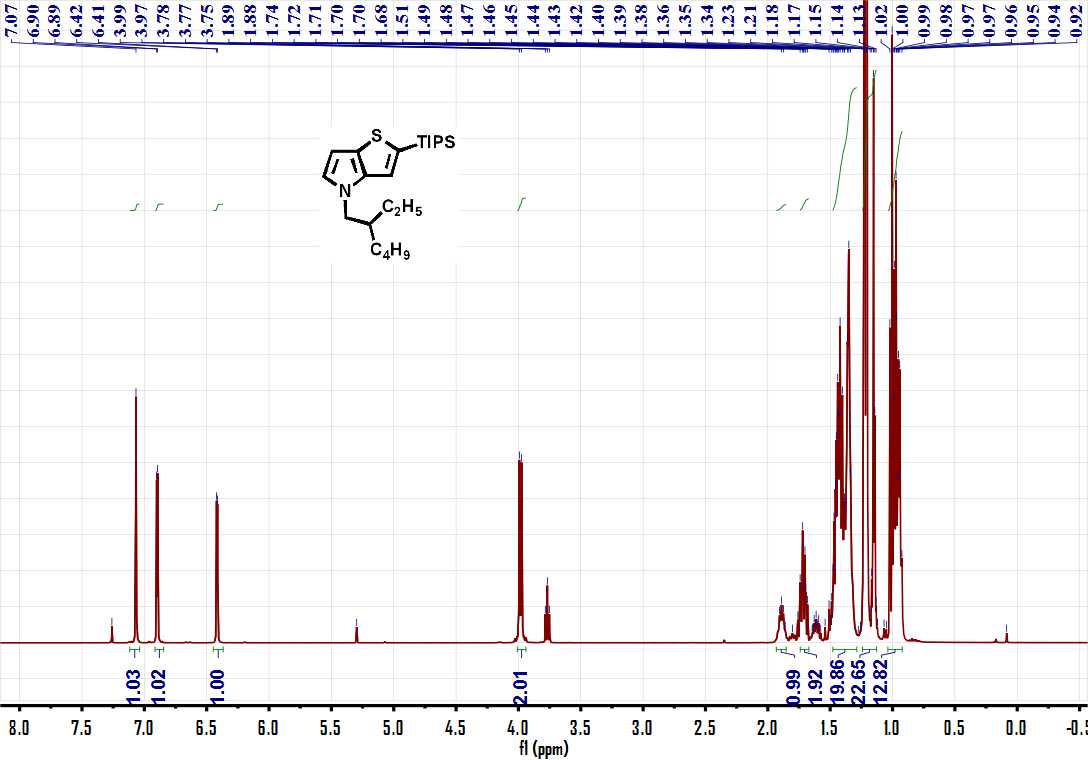


**Figure S8**. ^1^H NMR spectrum of Compound **5** in CDCl_3_.


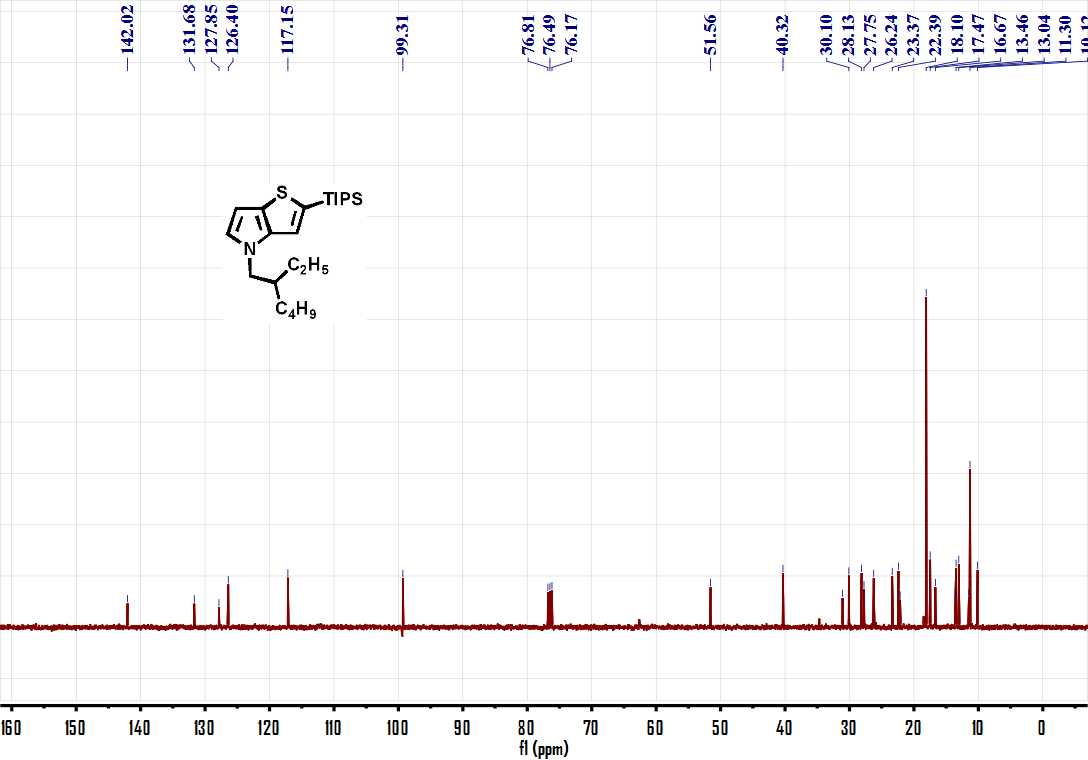


**Figure S9**. ^13^C NMR spectrum of Compound **5** in CDCl_3_.


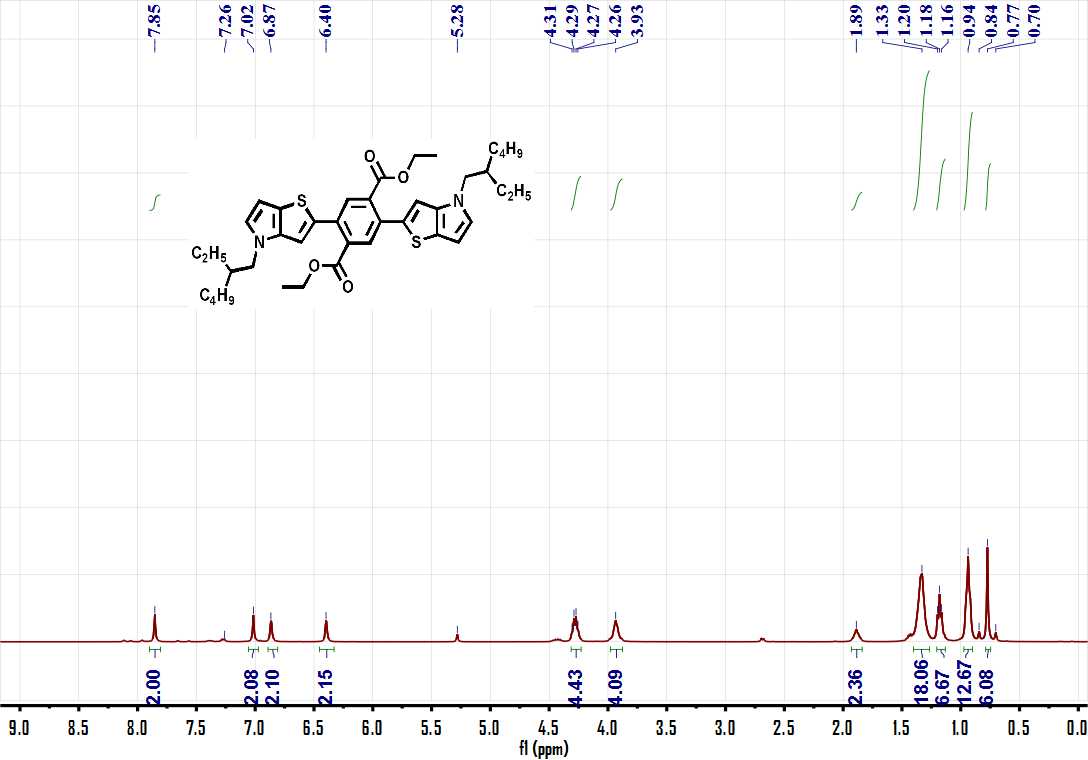


**Figure S10**. ^1^H NMR spectrum of compound **8** in CDCl_3_.


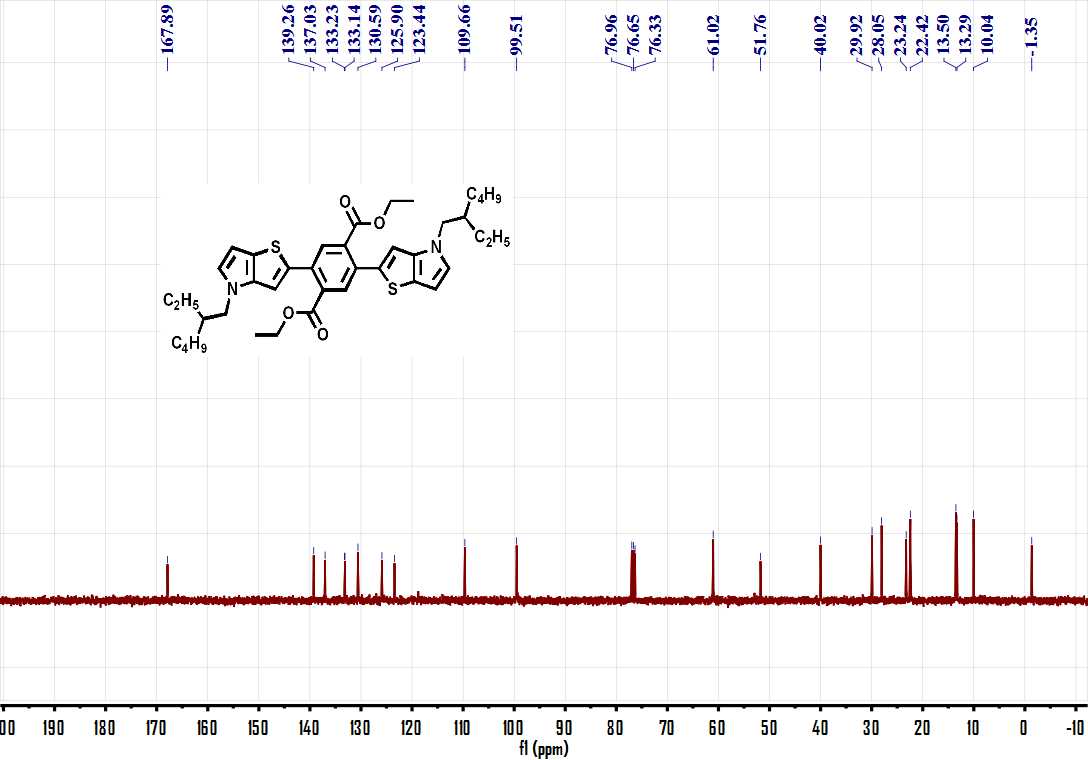


**Figure S11**. ^13^C NMR spectrum of compound **8** in CDCl_3_.


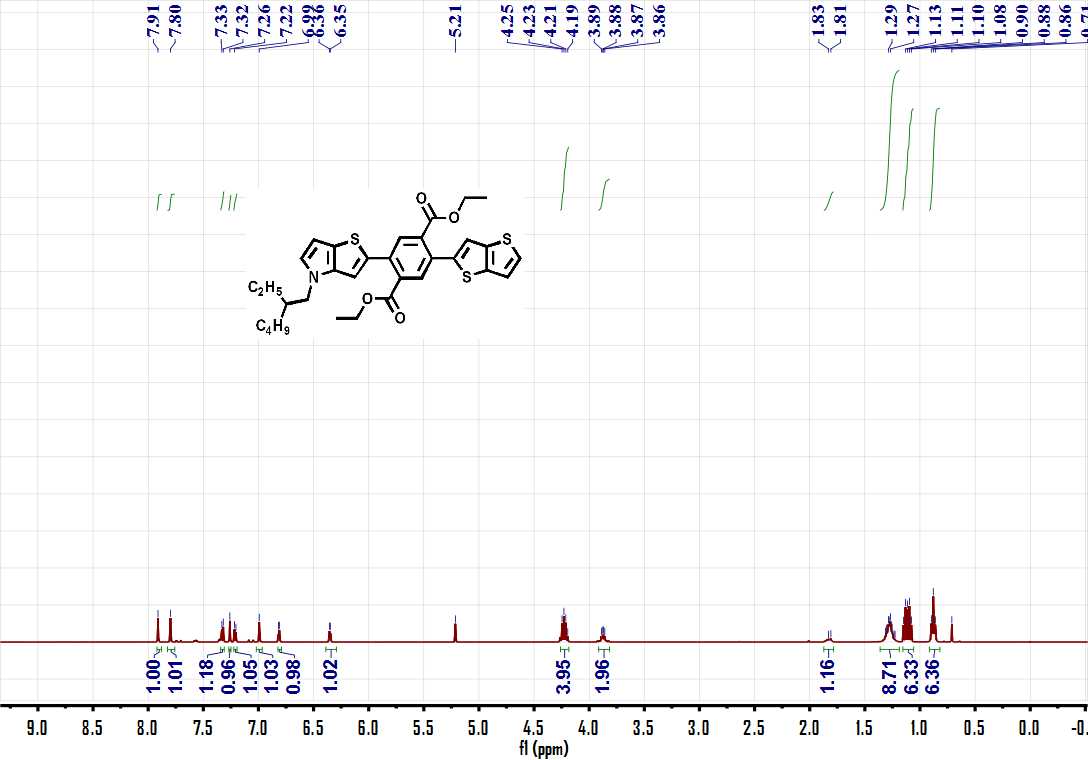


**Figure S12**. ^1^H NMR spectrum of compound **9** in CDCl_3_.

**
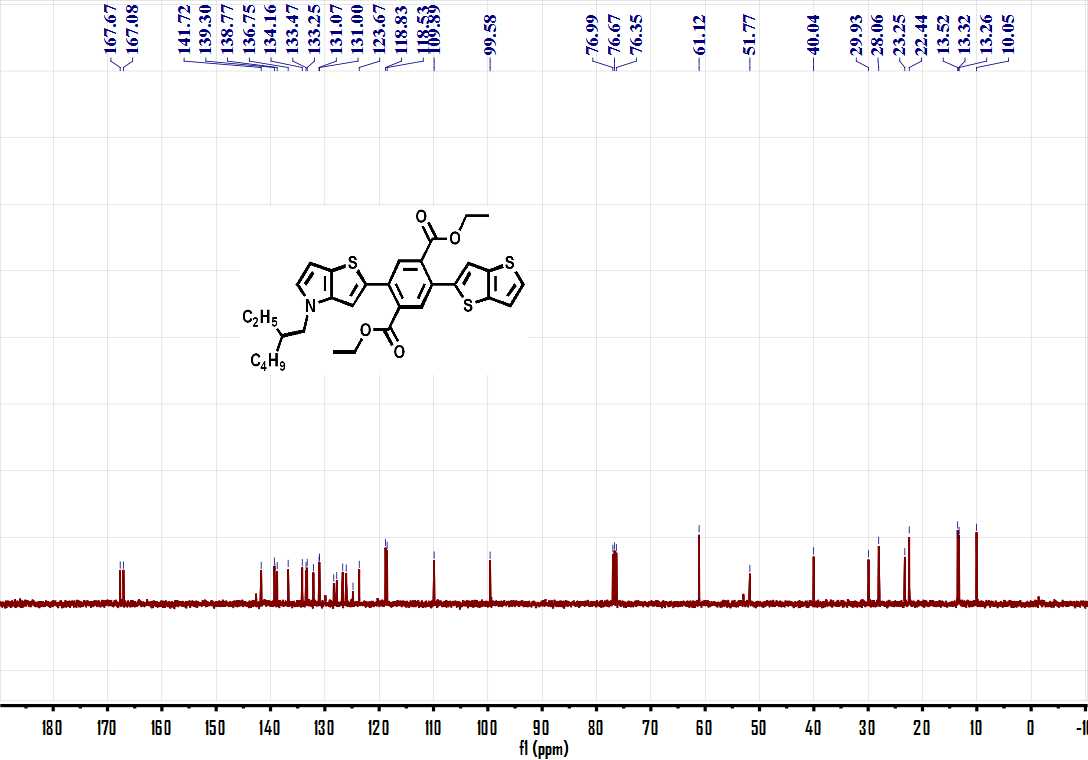
**

**Figure S13**. ^13^C NMR spectrum of compound **9** in CDCl_3_.


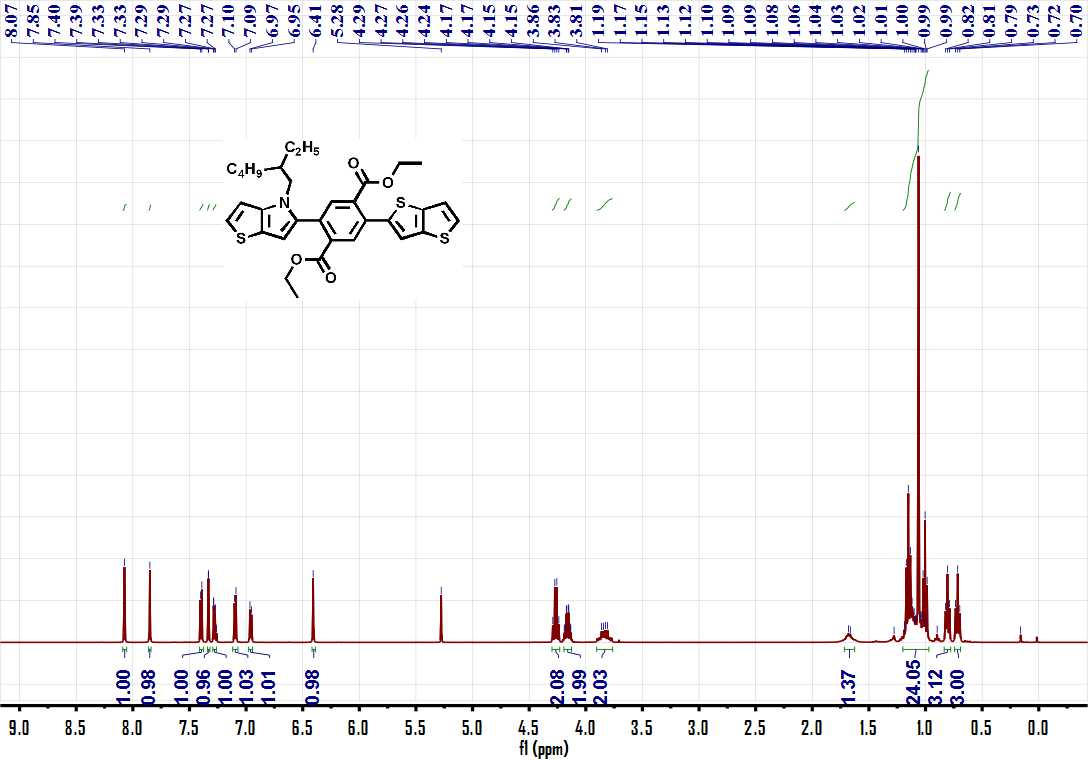


**Figure S14**. ^1^H NMR spectrum of compound **10** in CDCl_3_.


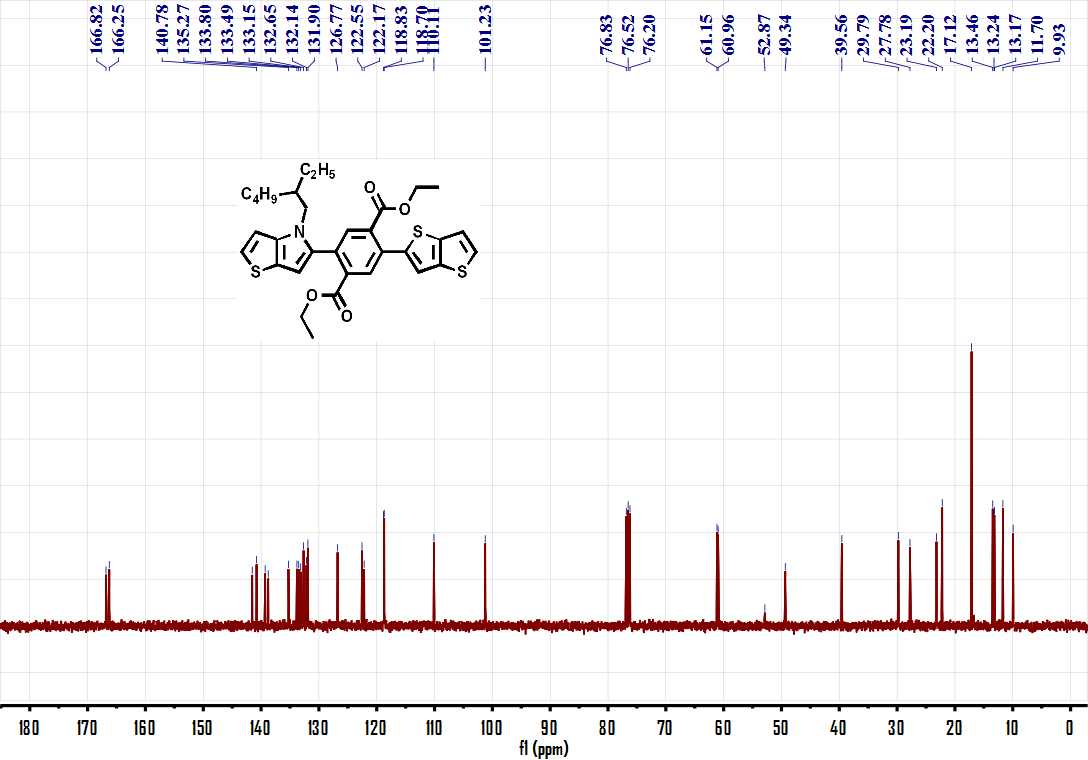


**Figure S15**. ^13^C NMR spectrum of compound **10** in CDCl_3_.

**
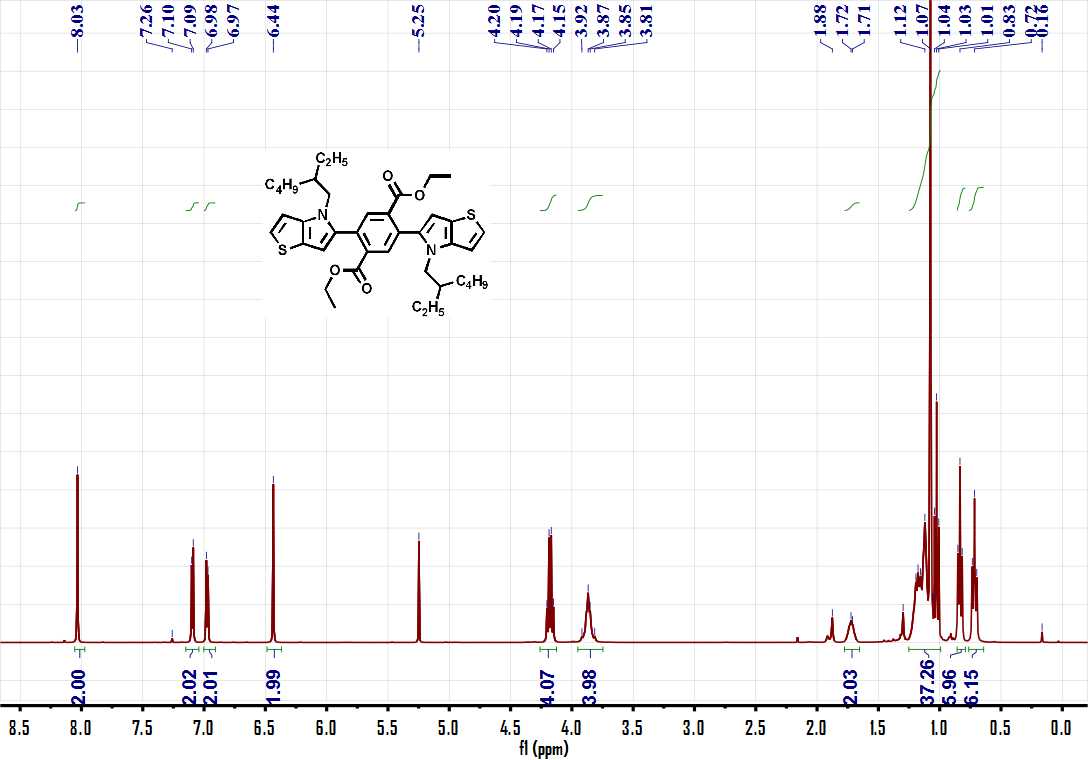
**

**Figure S16**. ^1^H NMR spectrum of compound **11** in CDCl_3_.


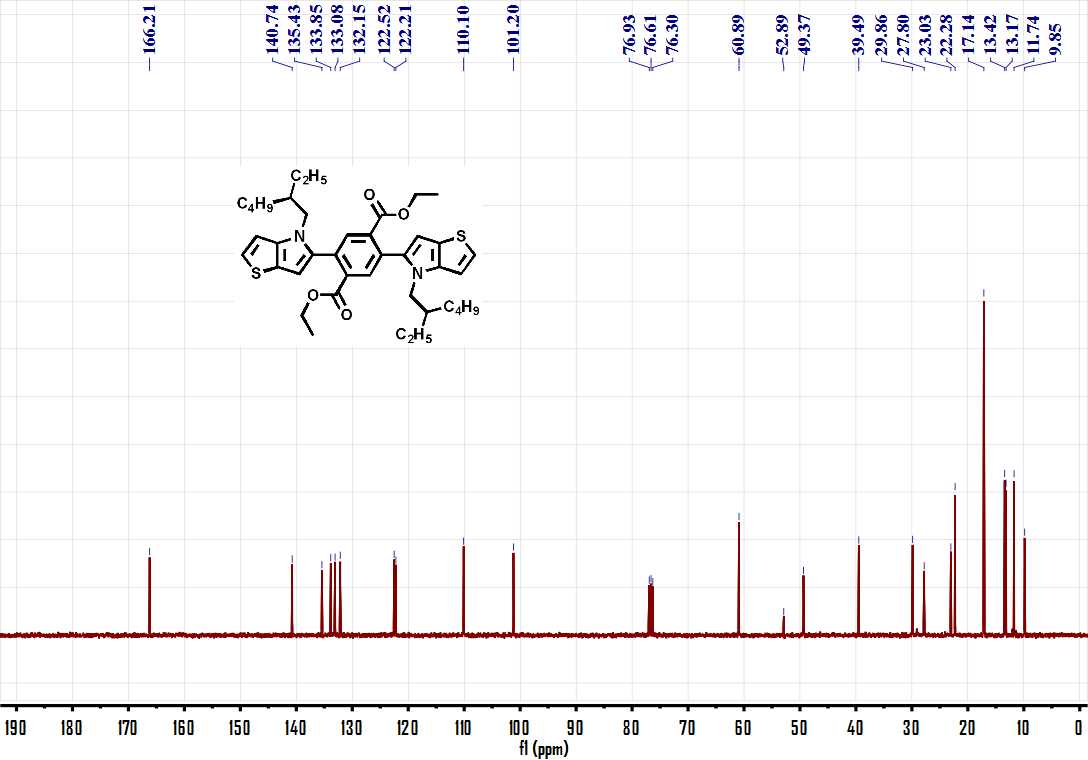


**Figure S17**. ^13^C NMR spectrum of compound **11** in CDCl_3_.

**
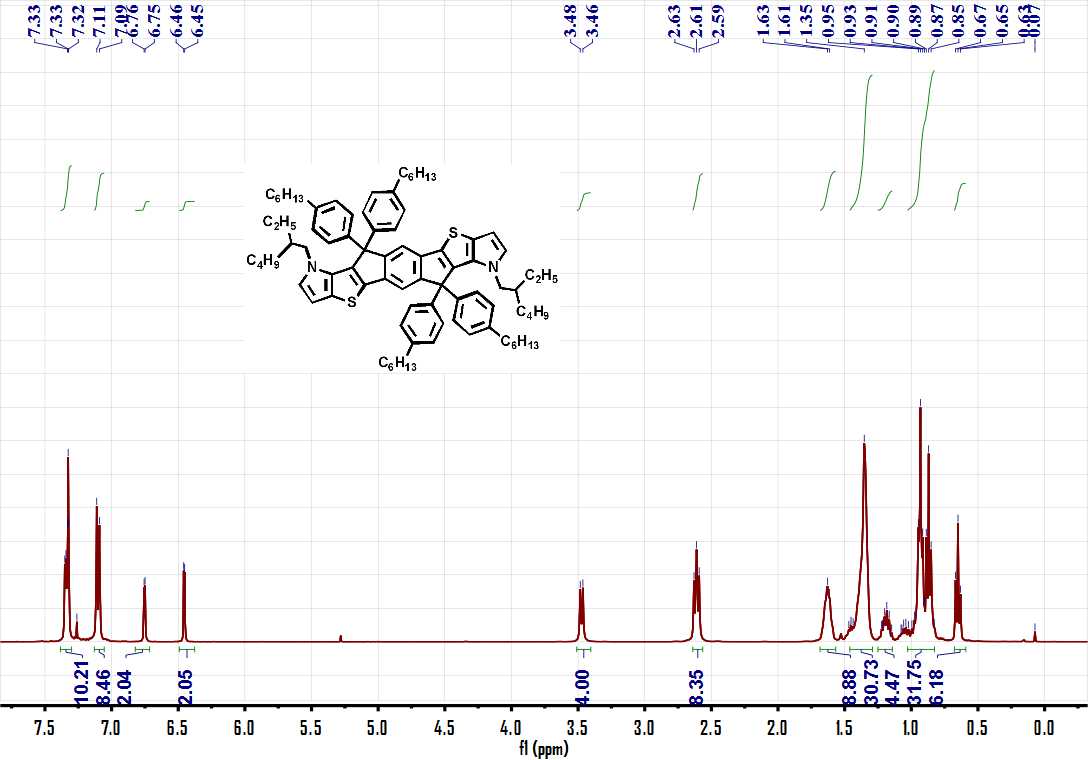
**

**Figure S18**. ^1^H NMR spectrum of compound **12** in CDCl_3_.


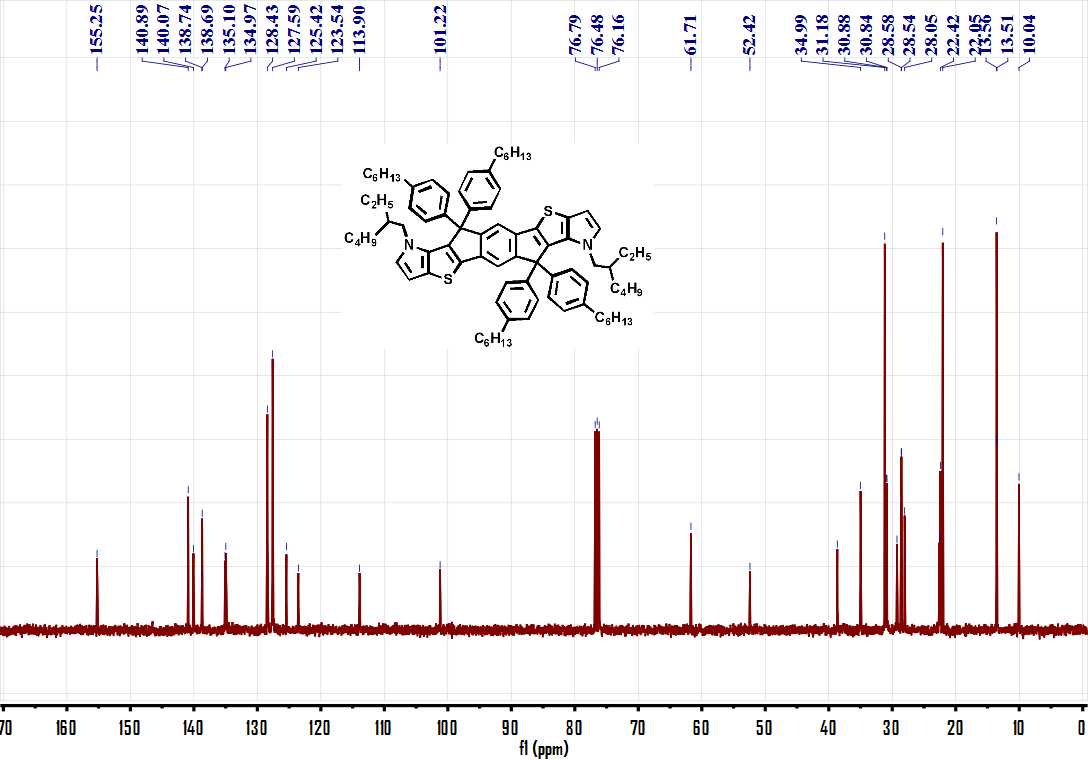


**Figure S19**. ^13^C NMR spectrum of compound **12** in CDCl_3_.


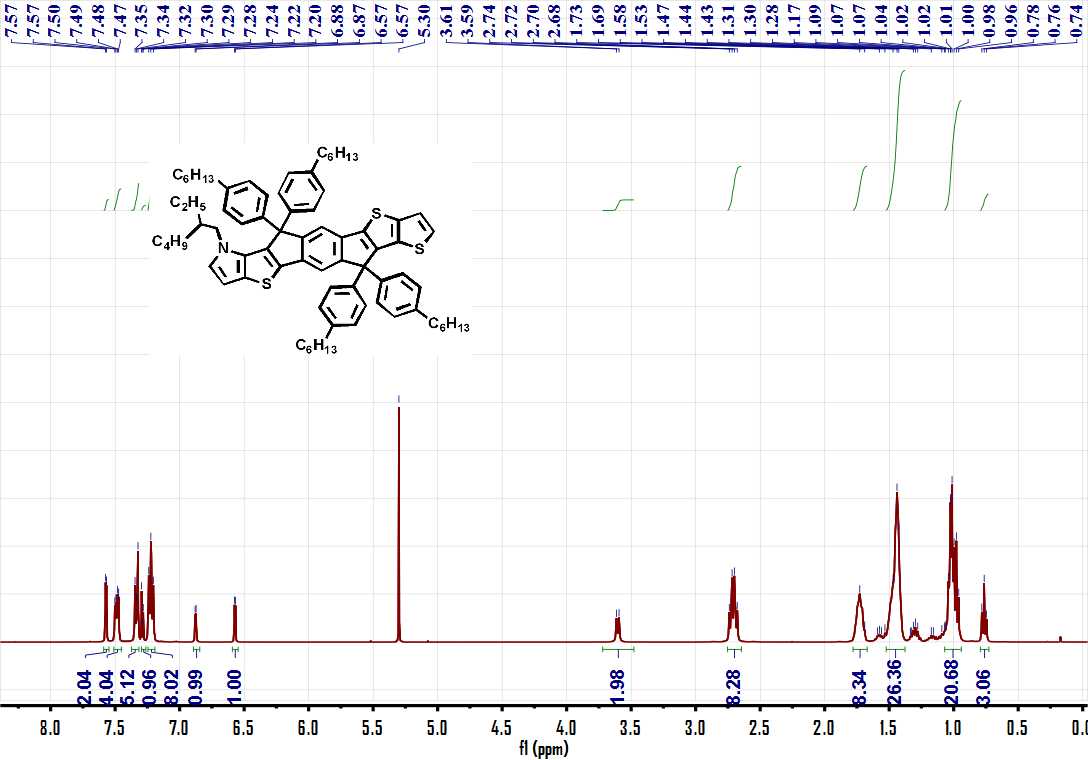


**Figure S20**. ^1^H NMR spectrum of compound **13** in CDCl_3_.


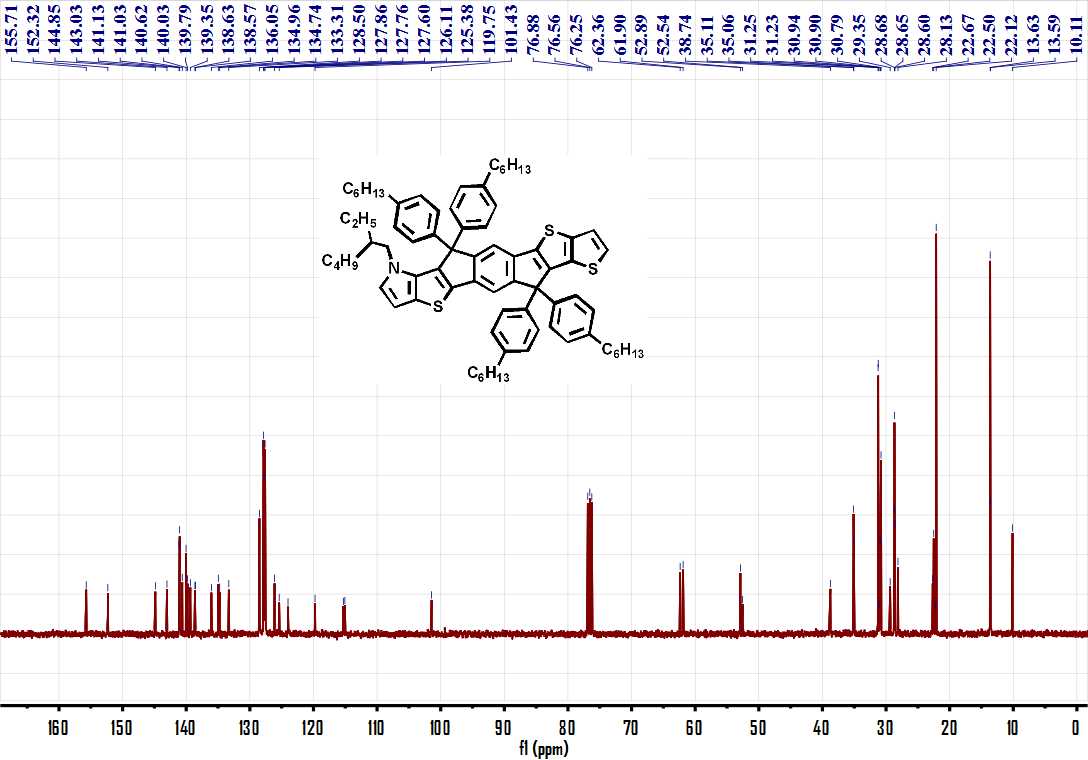


**Figure S21**. ^13^C NMR spectrum of compound **13** in CDCl_3_.


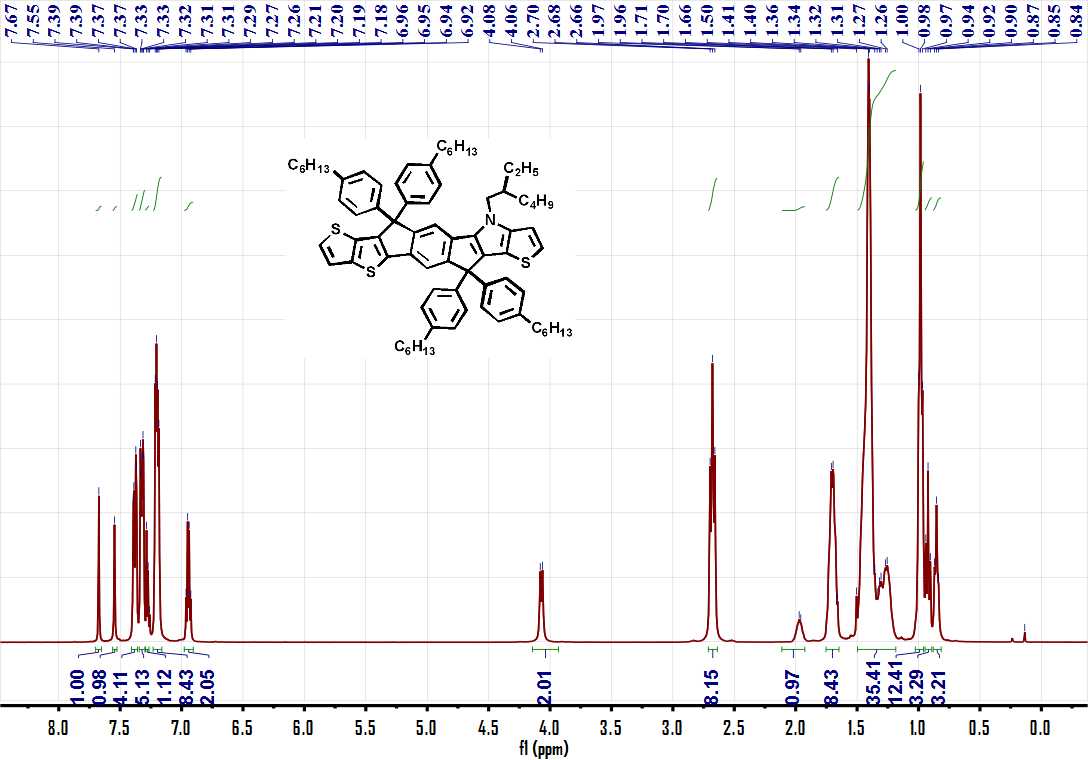


**Figure S22**. ^1^H NMR spectrum of compound **14** in CDCl_3_.


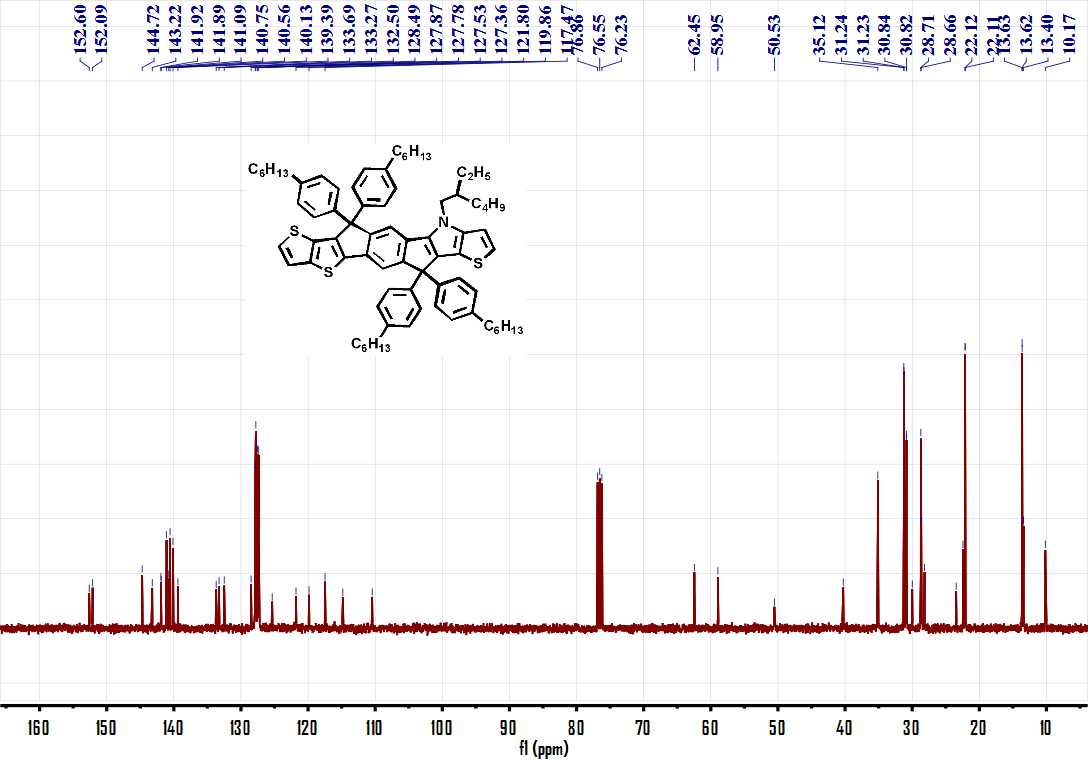


**Figure S23**. ^13^C NMR spectrum of compound **14** in CDCl_3_.


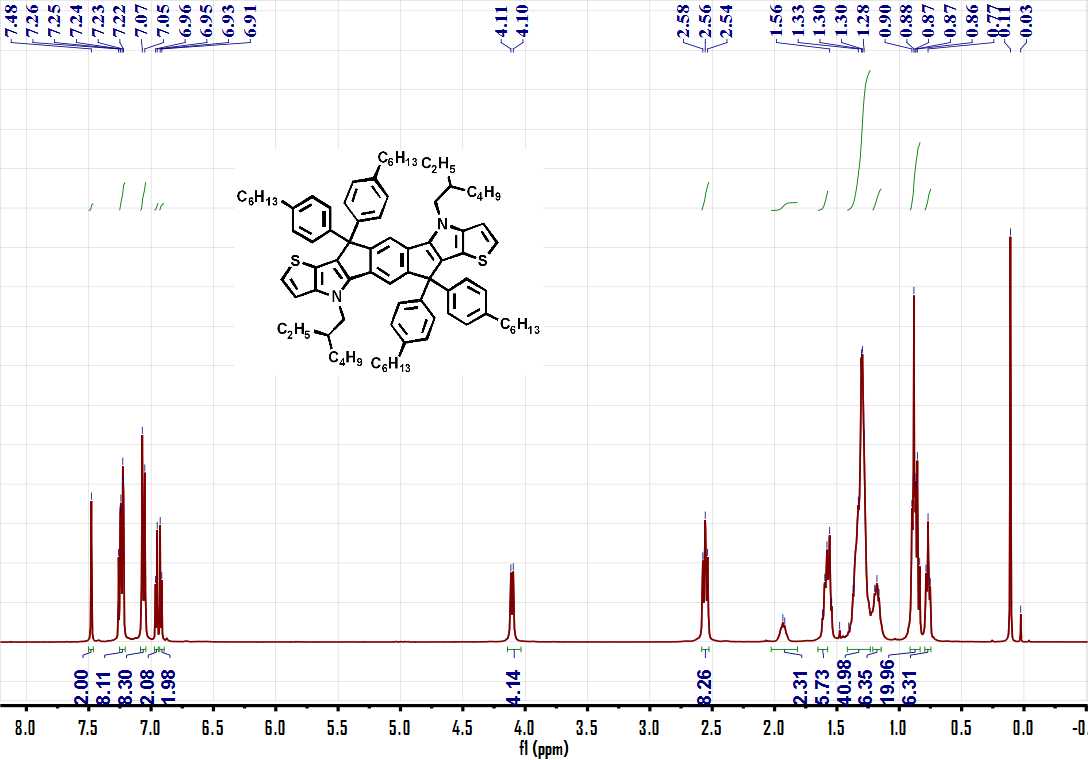


**Figure S24**. ^1^H NMR spectrum of compound **15** in CDCl_3_.


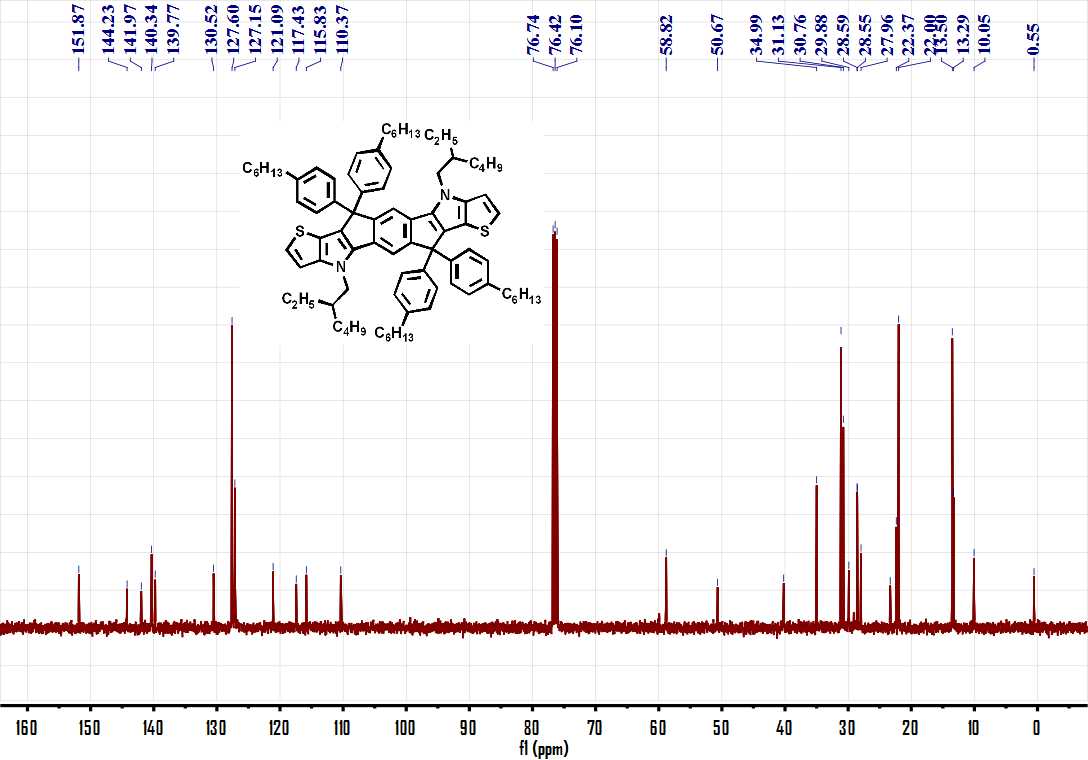


**Figure S25**. ^13^C NMR spectrum of compound **15** in CDCl_3_.


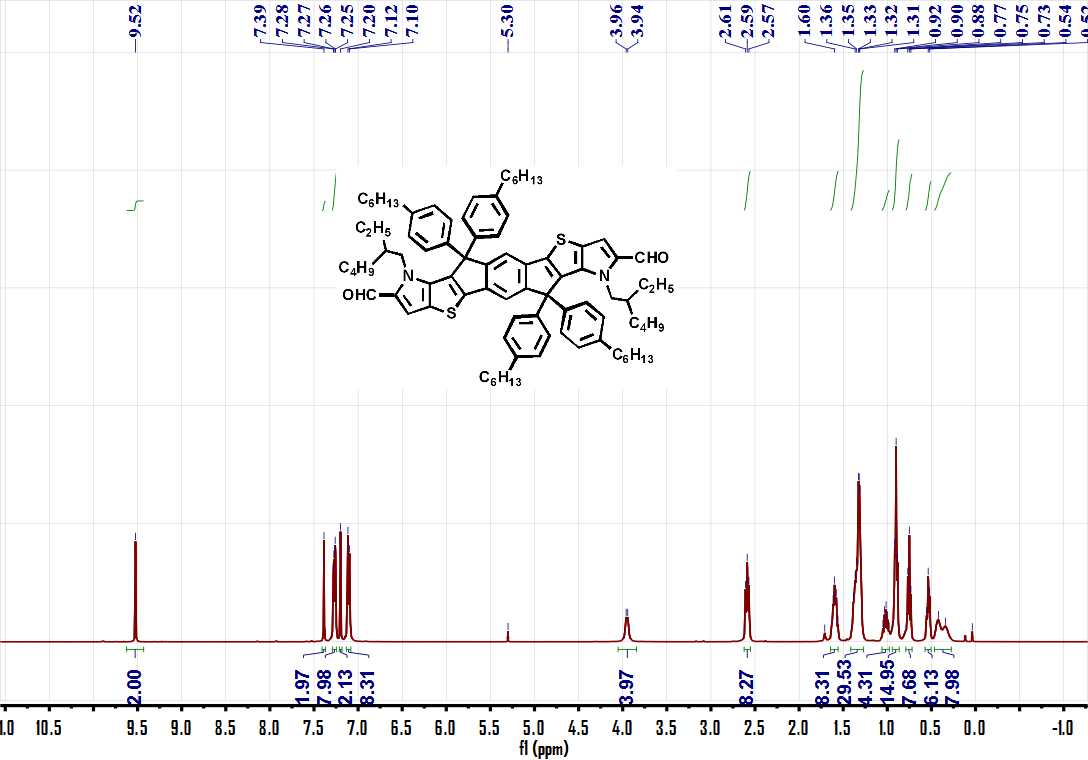


**Figure S26**. ^1^H NMR spectrum of compound **16** in CDCl_3_.


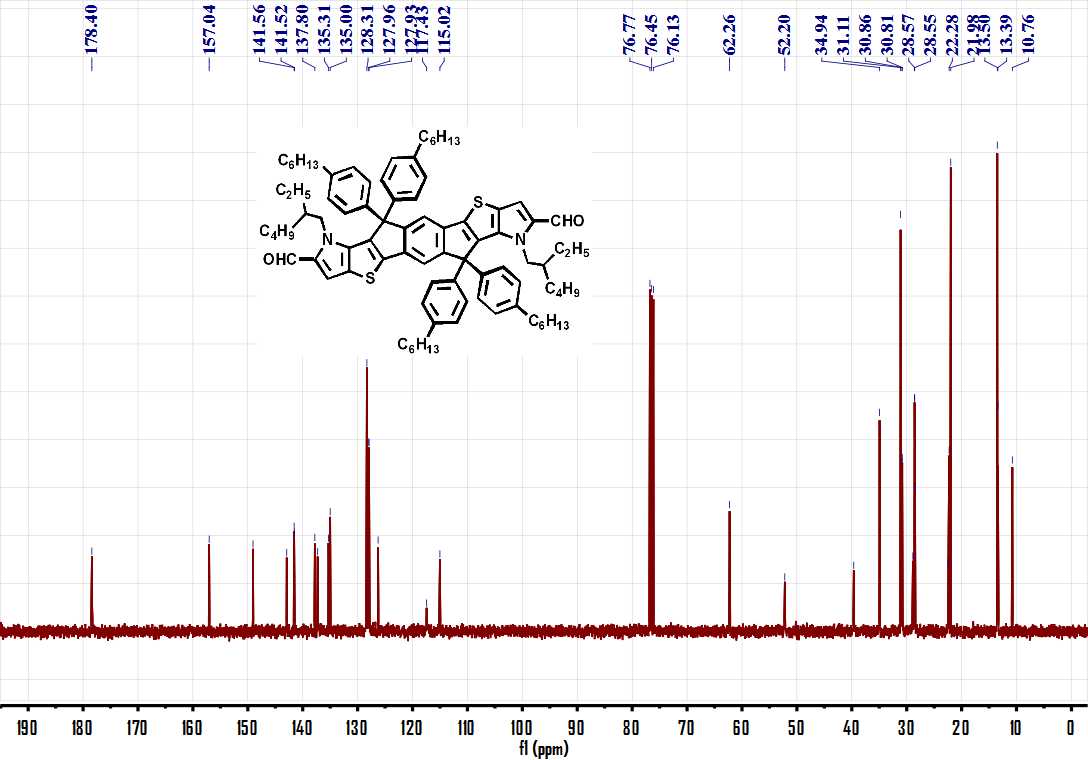


**Figure S27**. ^13^C NMR spectrum of compound **16** in CDCl_3_.


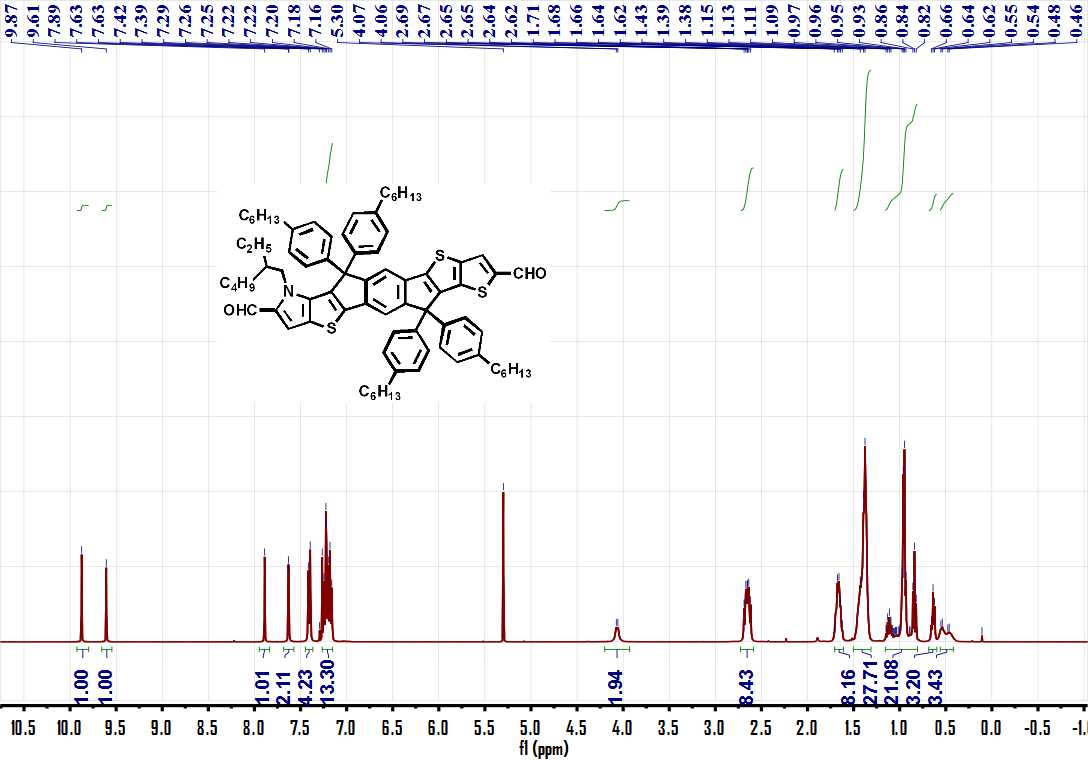


**Figure S28**. ^1^H NMR spectrum of compound **17** in CDCl_3_.


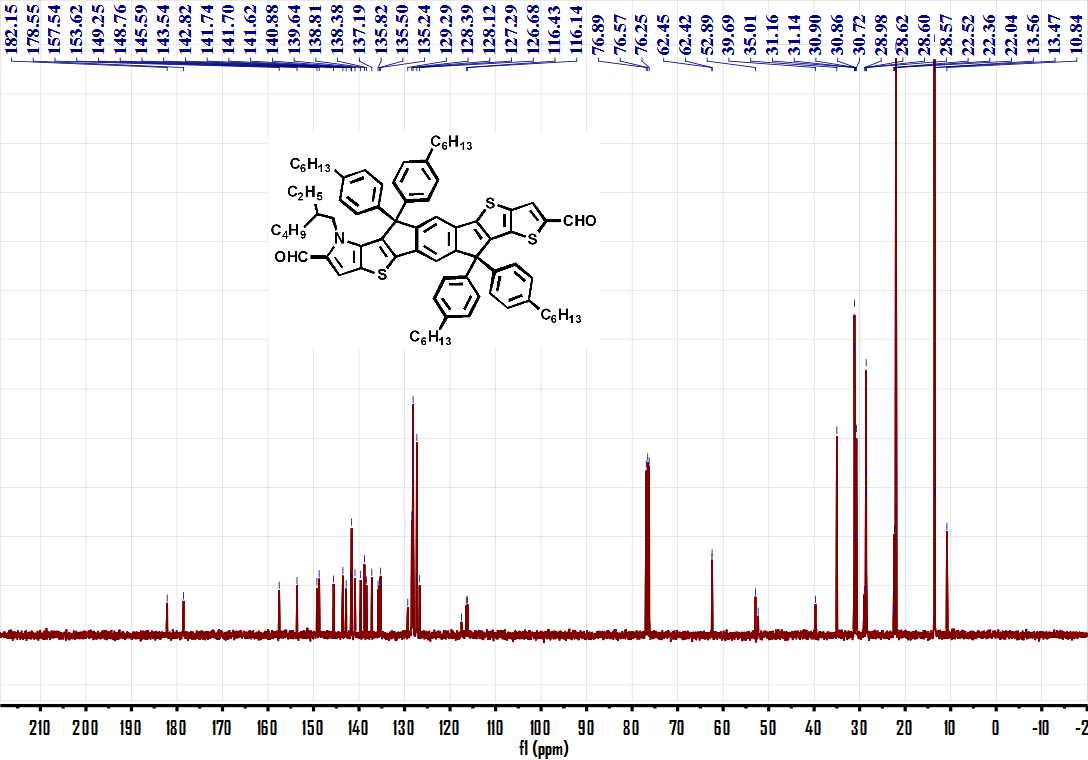


**Figure S29**. ^13^C NMR spectrum of compound **17** in CDCl_3_.


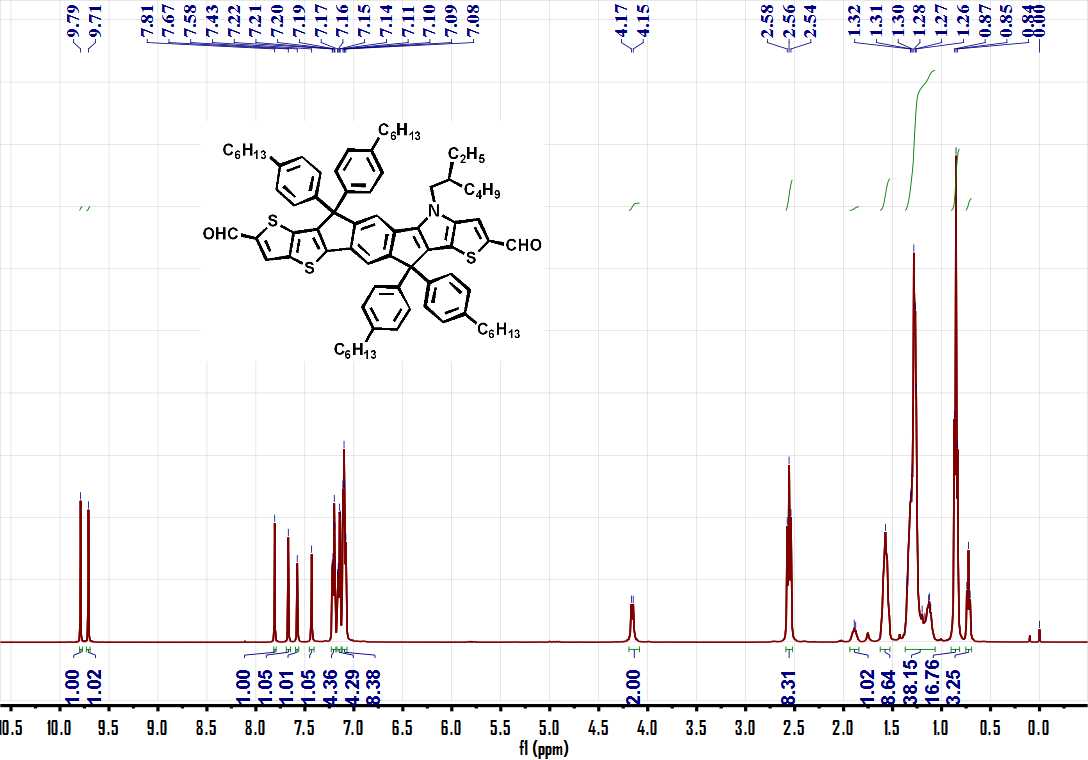


**Figure S30**. ^1^H NMR spectrum of compound **18** in CDCl_3_.


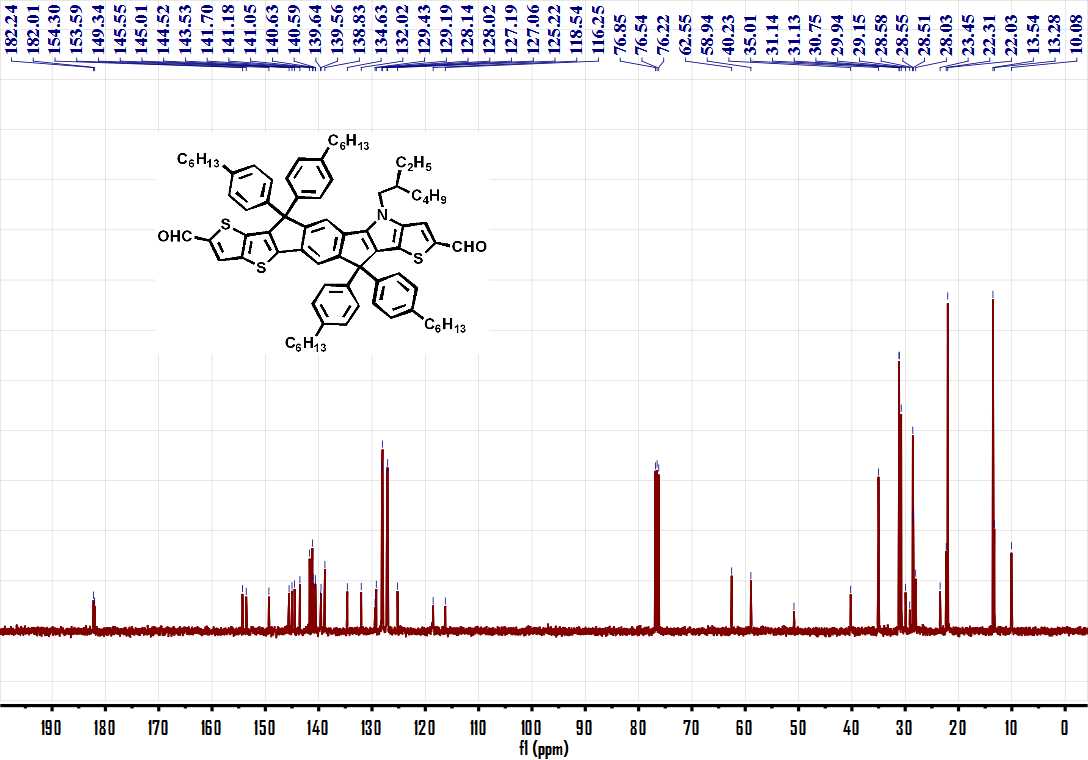


**Figure S31**. ^13^C NMR spectrum of compound **18** in CDCl_3_.


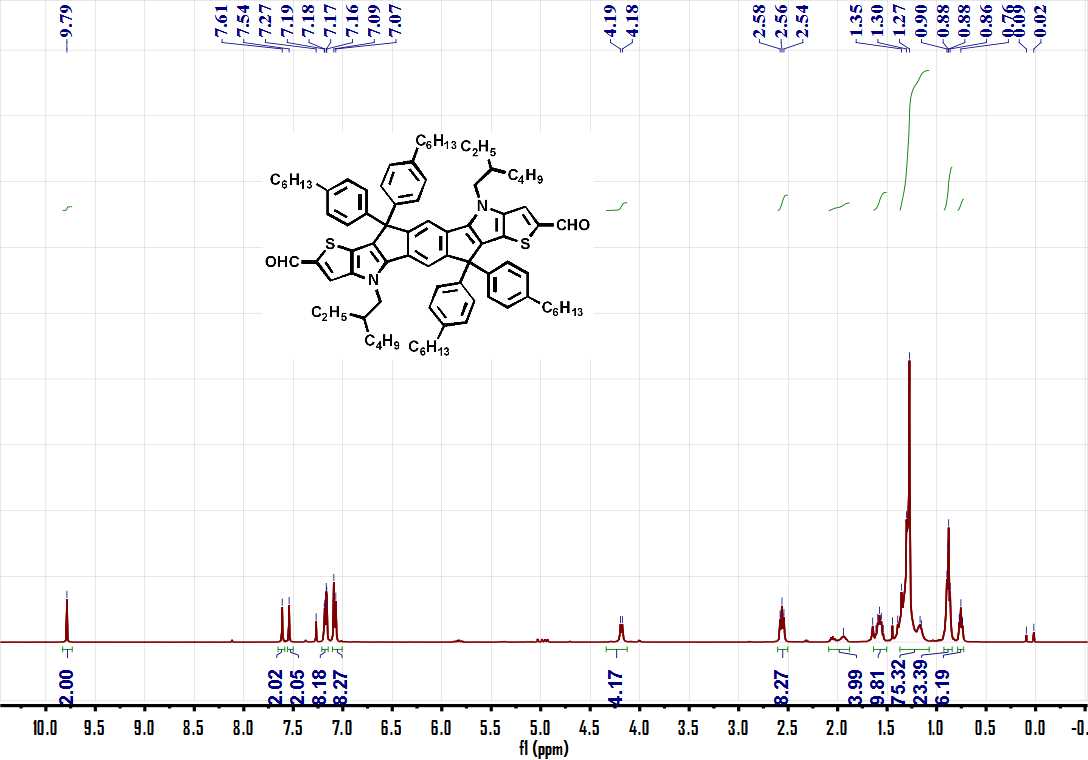


**Figure S32**. ^1^H NMR spectrum of compound **19** in CDCl_3_.


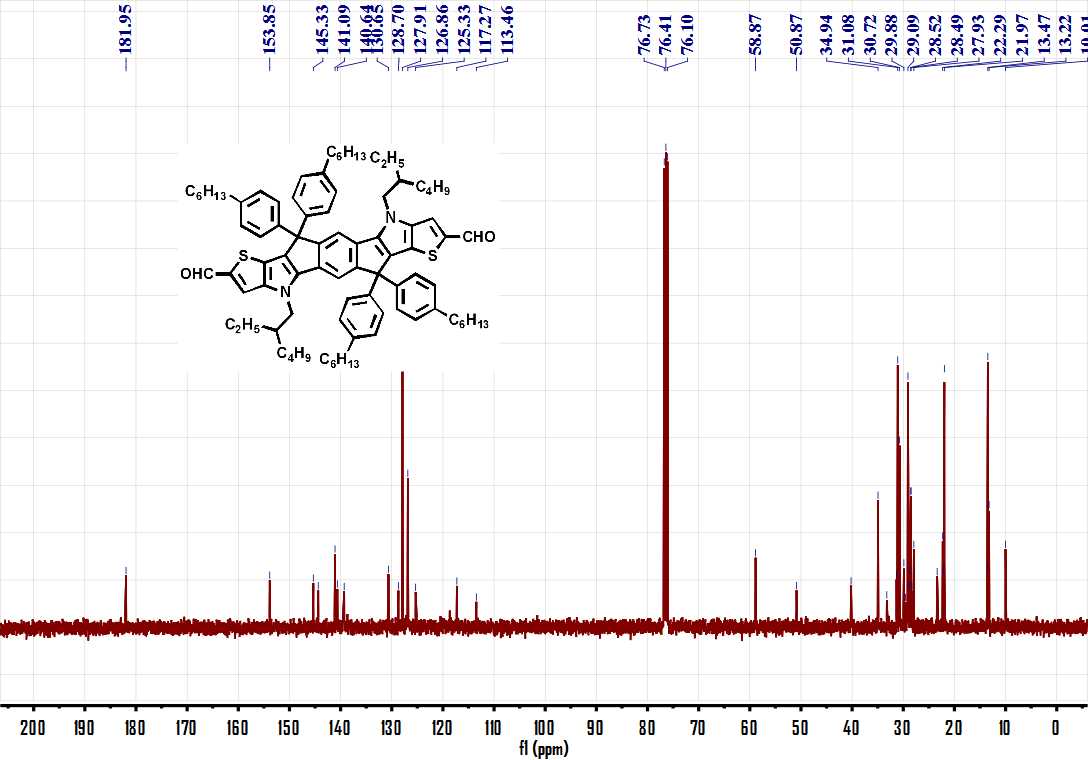


**Figure S33**. ^13^C NMR spectrum of compound **19** in CDCl_3_.


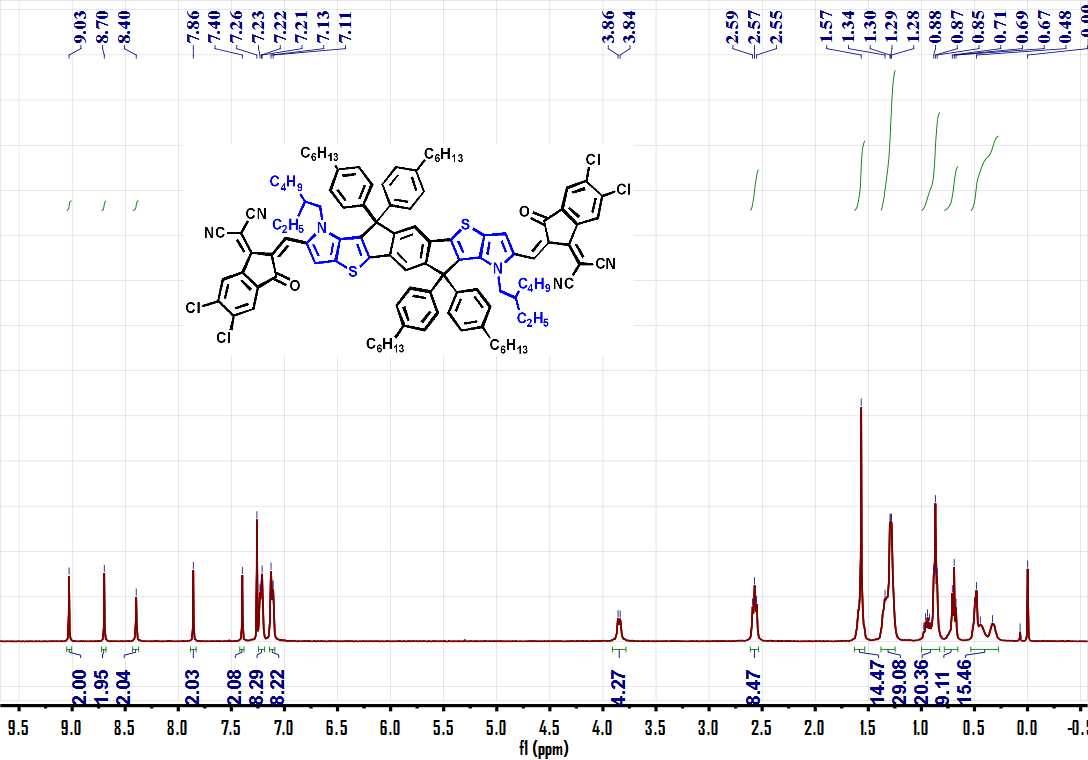


**Figure S34**. ^1^H NMR spectrum of **ThPy1** in CDCl_3_.


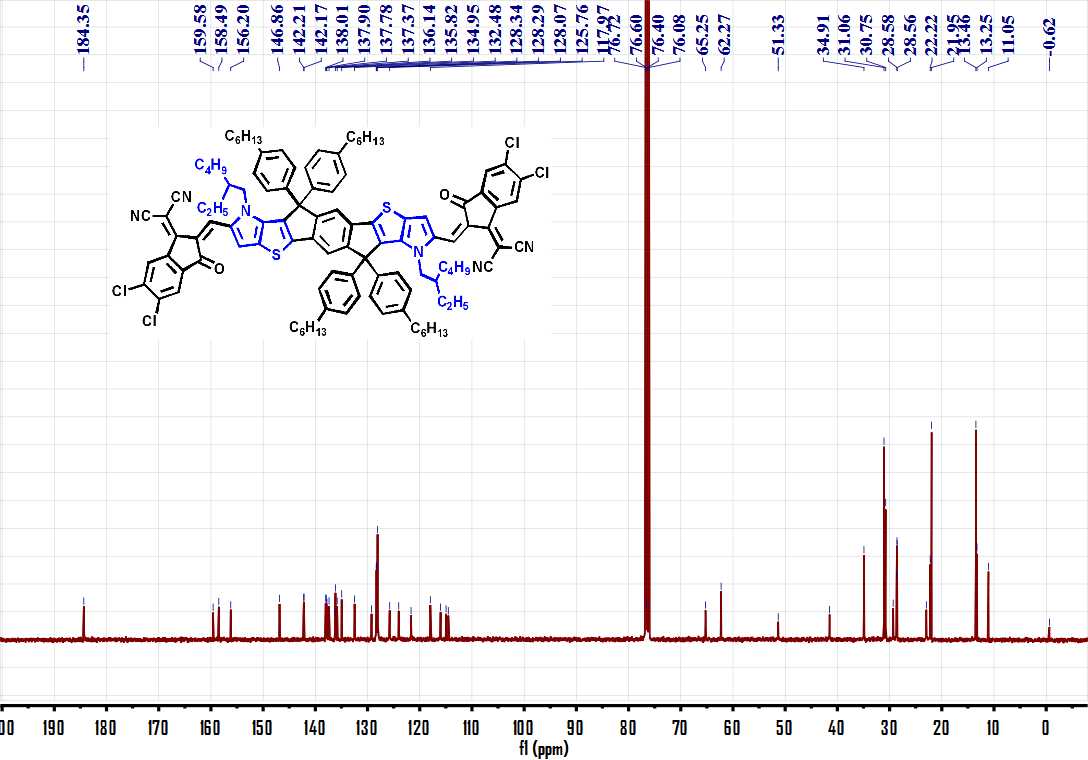


**Figure S35**. ^13^C NMR spectrum of **ThPy1** in CDCl_3_.


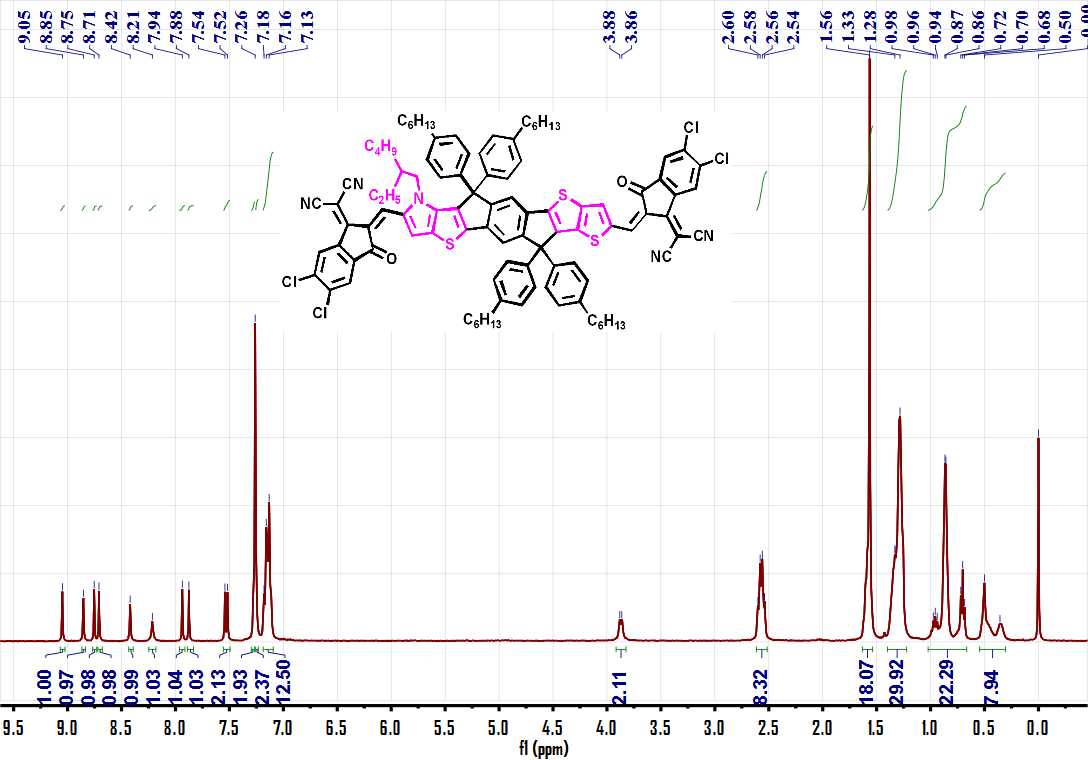


**Figure S36**. ^1^H NMR spectrum of **ThPy2** in CDCl_3_.

**
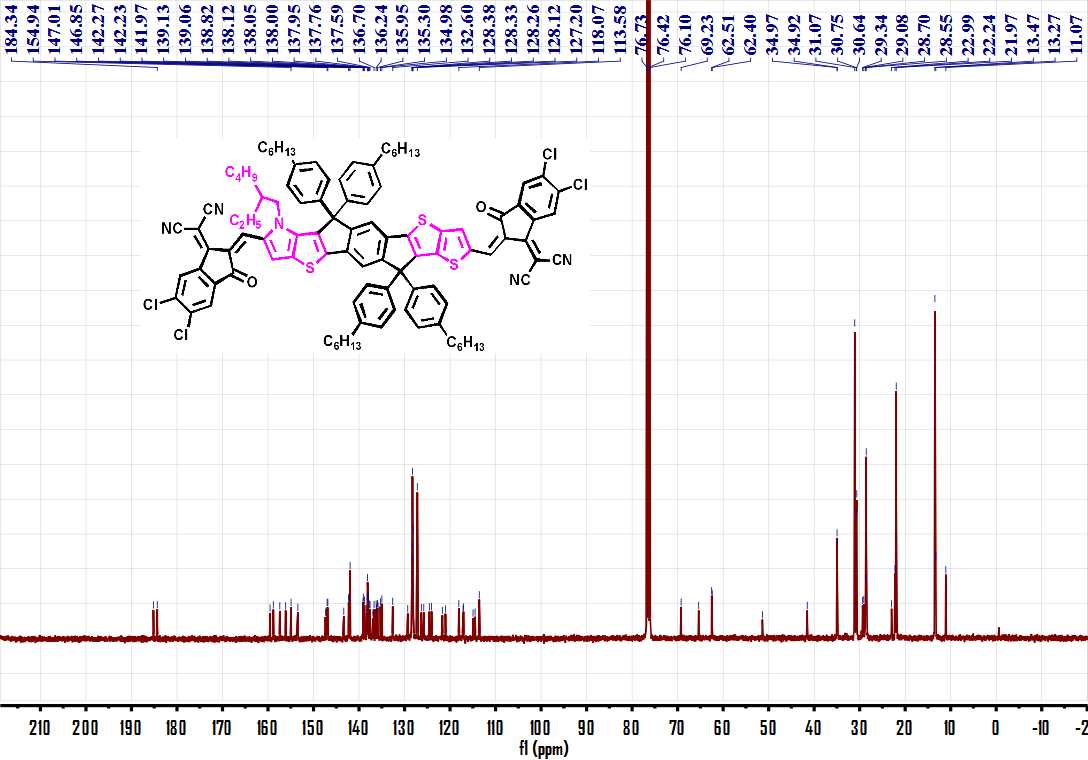
**

**Figure S37**. ^13^C NMR spectrum of **ThPy2** in CDCl_3_.


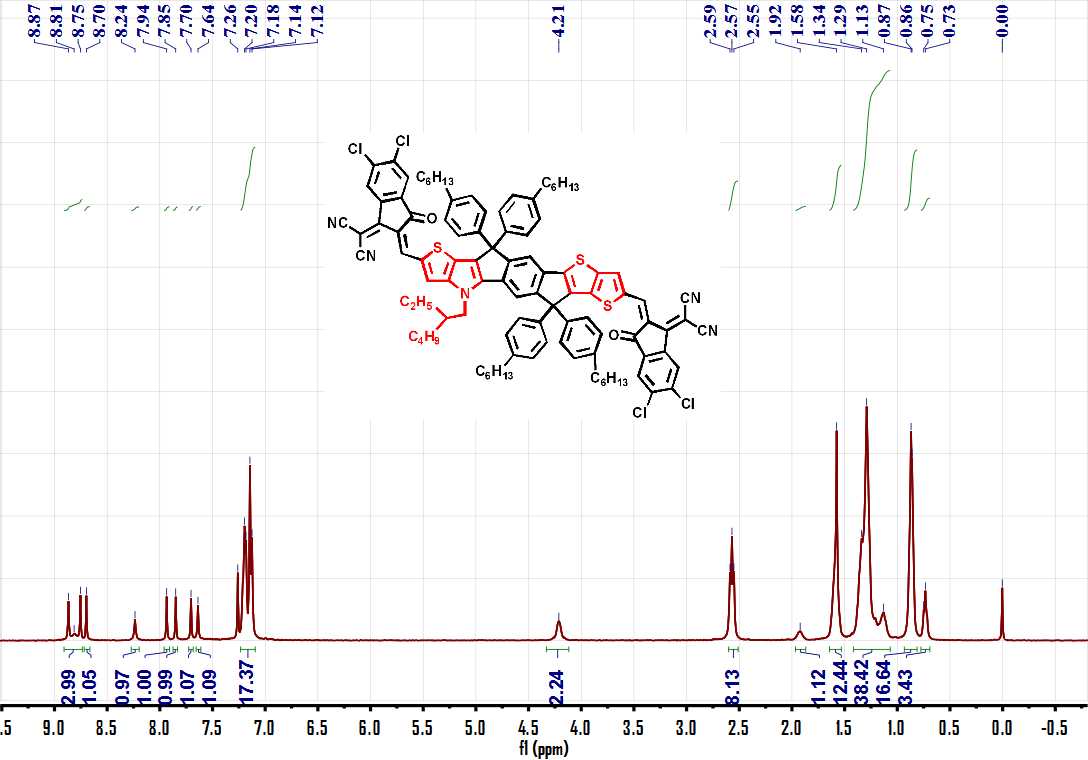


**Figure S38**. ^1^H NMR spectrum of **ThPy3** in CDCl_3_.


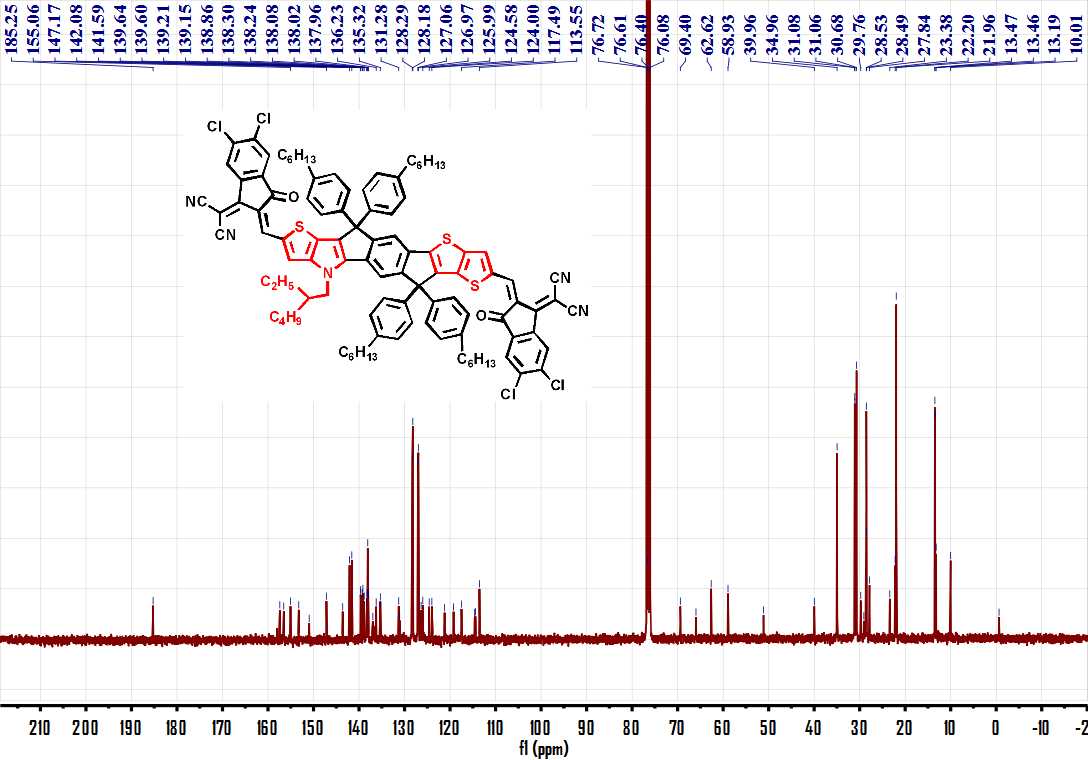


**Figure S39**. ^13^C NMR spectrum of **ThPy3** in CDCl_3_.


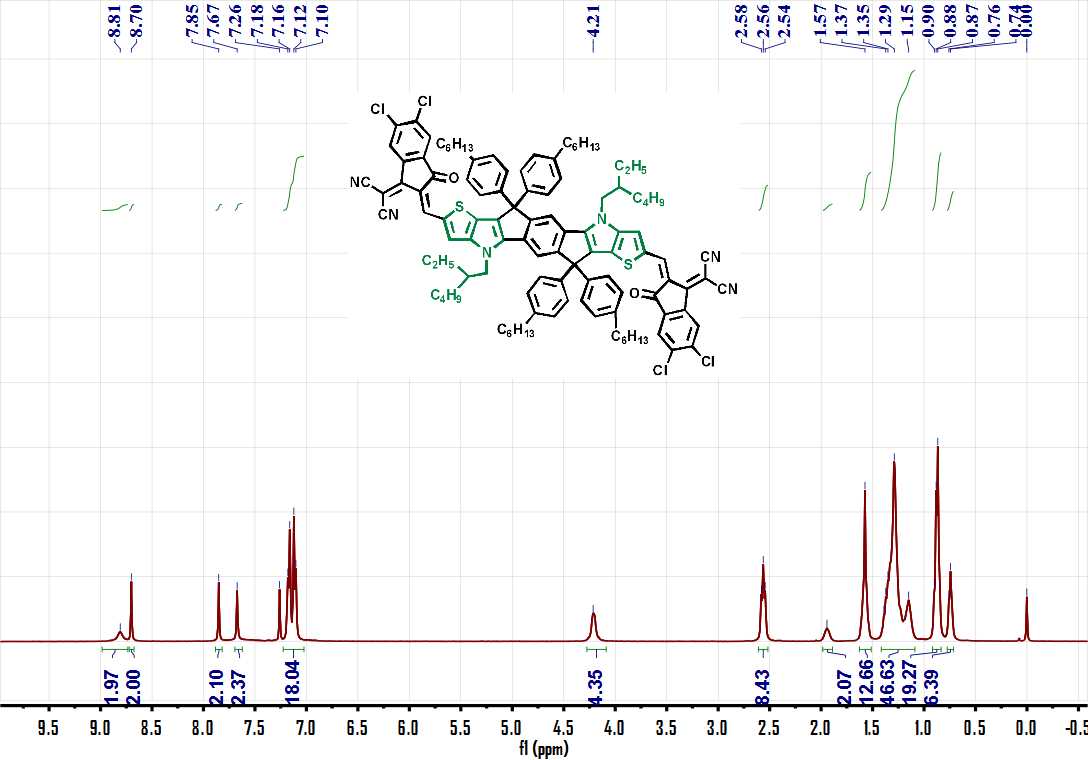


**Figure S40**. ^1^H NMR spectrum of **ThPy4** in CDCl_3_.


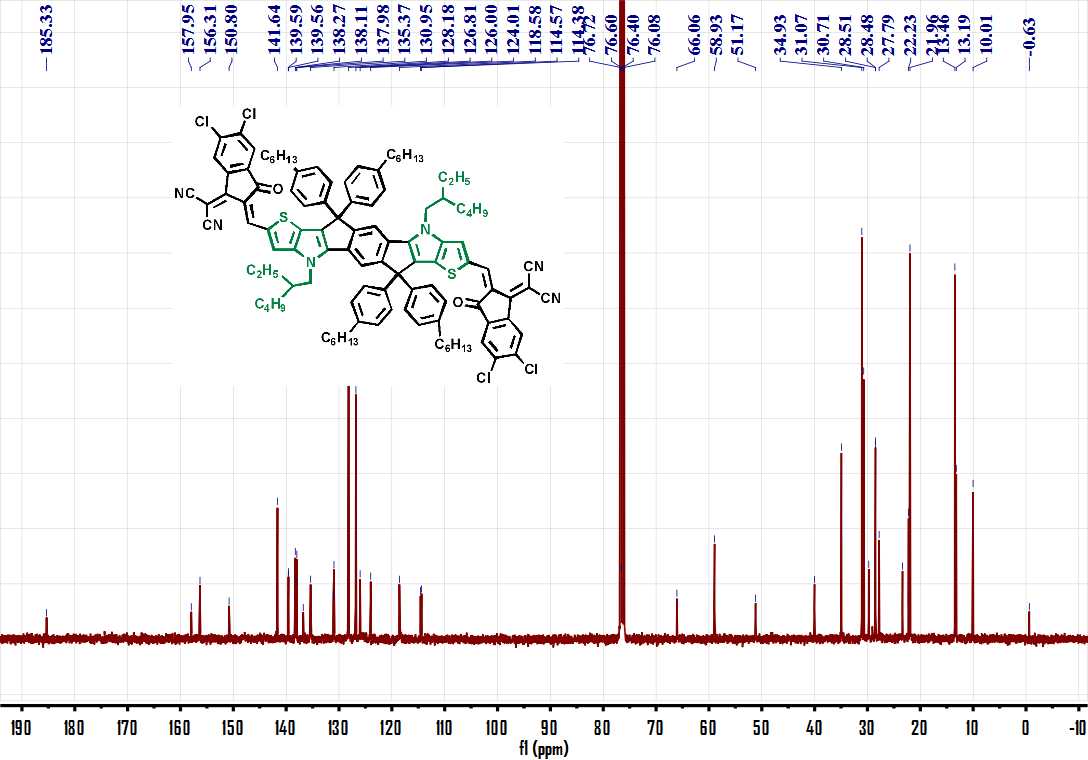


**Figure S41**. ^13^C NMR spectrum of **ThPy4** in CDCl_3_.

**Figure S42**. MS spectrum (MALDI-TOF) of ThPy1.

**Figure S43**. MS spectrum (MALDI-TOF) of ThPy2.

**Figure S44**. MS spectrum (MALDI-TOF) of ThPy2.

**Figure S45**. MS spectrum (MALDI-TOF) of ThPy4.

References

[1] C. Bulumulla, R. Gunawardhana, R. N. Kularatne, M. E. Hill, G. T. McCandless, M. C. Biewer and M. C. Stefan, *ACS Appl. Mater. Interfaces* **2018**, *10*, 11818.
